# Alternative utrophin mRNAs contribute to phenotypic differences between dystrophin‐deficient mice and Duchenne muscular dystrophy

**DOI:** 10.1002/1873-3468.13099

**Published:** 2018-05-30

**Authors:** Kelly J. Perkins, Kay E. Davies

**Affiliations:** ^1^ Department of Physiology Anatomy and Genetics University of Oxford UK; ^2^ Sir William Dunn School of Pathology University of Oxford UK

**Keywords:** Duchenne muscular dystrophy, therapy, utrophin

## Abstract

Duchenne muscular dystrophy (DMD) is a fatal disorder caused by absence of functional dystrophin protein. Compensation in dystrophin‐deficient (*mdx*) mice may be achieved by overexpression of its fetal paralogue, utrophin. Strategies to increase utrophin levels by stimulating promoter activity using small compounds are therefore a promising pharmacological approach. Here, we characterise similarities and differences existing within the mouse and human utrophin locus to assist in high‐throughput screening for potential utrophin modulator drugs. We identified five novel 5′‐utrophin isoforms (A′,B′,C,D and F) in adult and embryonic tissue. As the more efficient utrophin‐based response in *mdx* skeletal muscle appears to involve independent transcriptional activation of conserved, myogenic isoforms (A′ and F), elevating their paralogues in DMD patients is an encouraging therapeutic strategy.

## Abbreviations

**5′RACE**, 5′ Rapid Amplification of cDNA Ends

**DMD**, Duchenne muscular dystrophy

**EMSA**, electrophoretic mobility shift assay

**ENCODE**, encyclopedia of DNA elements

**HS**, hypersensitivity site

**MAB**, mesoangioblast

**qRT‐PCR**, quantitative RT‐PCR

**RLM**, RNA ligase‐mediated

**TA**,*tibialis anterior*

**UtroF**, F‐utrophin antibody

Counteracting the absence of functional dystrophin in Duchenne muscular dystrophy (DMD) individuals *via* upregulation of its autosomal paralogue, utrophin 1, has been an appealing option for over two decades. The discovery and preliminary characterization of utrophin presented an ideal candidate to devise a replacement therapeutic strategy given its extensive protein sequence homology, size and functional properties with dystrophin, including association with the dystroglycan complex and F‐actin 2, 3, 4, 5. Furthermore, developmental mouse (*Utrn)* and human (*UTRN)* transcription precedes that of dystrophin, and as such is considered a fetal precursor 6. The latter is particularly evident during skeletal muscle development in mice and humans, where sarcolemmal *Utrn/UTRN* mRNAs rapidly decline upon dystrophin expression 7 resulting in postnatal confinement primarily to neuromuscular and myotendinous junctions 6, 8, 9. Indeed, utrophin has been shown to physiologically rescue dystrophin‐deficient *mdx* 10, 11 mouse skeletal muscle by varied means, including transgenic upregulation, viral delivery and oral compound administration (reviewed in 12). Consequently, discovery and exploitation of regulatory mechanisms that raise endogenous human utrophin mRNA levels are of great interest.

**Figure 1:**
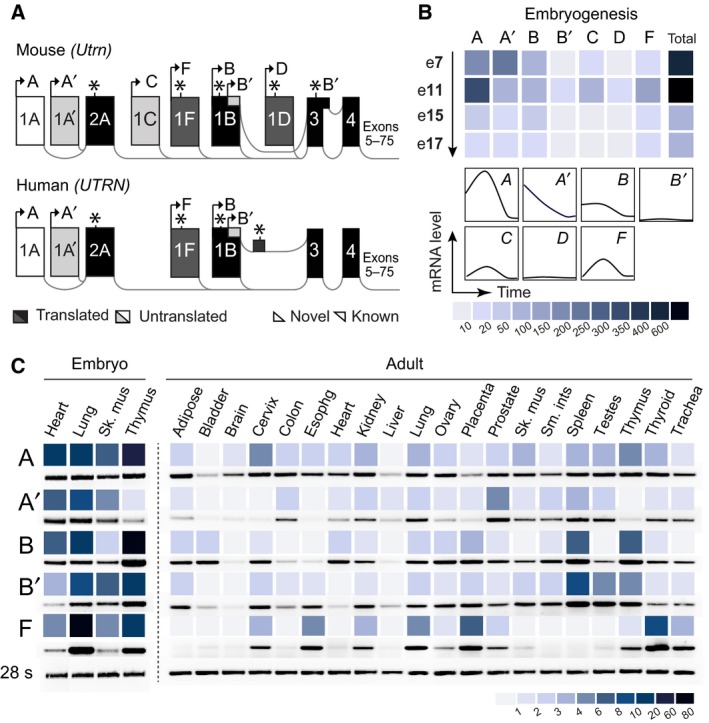
Identification and characterization of novel 5′ utrophin isoforms. 5′RACE of mouse and human RNA reveals novel utrophin exons within the large genomic interval between utrophin exon 1A and 3. (A) Schematic of (upper) mouse and (lower) human utrophin loci, with exon splicing patterns (grey lines) and type as denoted (untranslated exons: white or light grey; partially or completely translated exons: black or dark grey; lighter coloured exons are novel); distance is not to scale. Arrows signify exons containing transcription start sites with splicing patterns denoted to at least the first common exon (exon 4/3 for mouse/human respectively) represented. Asterisks indicate locations of translation start sites. (B) Embryonic mouse *Utrn* transcripts display distinctive profiles, indicating regulatory differences. Quantitative RT‐PCR (qRT‐PCR) of *Utrn*‐A/A′, *Utrn*‐B/B′ and *Utrn*‐C,‐D and ‐F isoforms at embryonic stages e7–17 as indicated. (C) Human *UTRN* mRNAs show isoform variability in their pre and postnatal tissue distribution. Left panel: qRT‐PCR analysis of human isoforms in fetal tissue, right panel: semiquantitative (sq)RT‐PCR of RNA sourced from 20 adult tissues, where values are arbitrary units standardised to 28 s cDNA. The colour scales provided in (B) and (C) represent values obtained for each sample. Human adult tissue sqRT‐PCR products were quantified on agarose gels, with representative bands provided below. Numerical values obtained for all sample sets are provided in Table S1. sk. musc, skeletal muscle; oesophg, oesophagus; sm. ints, small intestine.

At the transcription level, two conserved, independently regulated utrophin promoters designated A 13 and B 14 transcribe full‐length mRNAs that splice to exon 3 and differ only at their 5′ ends (Fig. 1A). Postnatal A‐utrophin protein localises to the adult neuromuscular junction, peripheral nerve and arterioles and, like dystrophin‐deficient *mdx* mice*,* DMD patients frequently show retained postnatal sarcolemmal A‐utrophin 15, 16, 17. Given its upregulation and extrajunctional properties in *mdx* and to an extent in DMD striated muscle, *UTRN*‐A is the favoured promoter‐based therapeutic target for current strategies, as B‐utrophin 17 remains restricted to endomysial capillaries and blood vessels 15, 18.

Interestingly, the relatively mild *mdx* mouse phenotype appears conferred *via* postnatal mechanisms apparently absent in DMD patients, where limb muscle degeneration is effectively countered by reversion to fetal myogenic programming, including *Utrn* re‐expression 19, 20. Furthermore, mice deficient in both full‐length dystrophin and utrophin (*dko*) are a closer DMD phenocopy 21, 22, implicating the regulatory control of utrophin in contributing to this *mdx*/DMD phenotypic disparity.

Given that the protein localization of A‐ and B‐utrophin appears conserved within skeletal muscle 15, 16, 17, we postulated that mouse and human loci respond differently to counteract dystrophin deficiency by transcriptional regulation of alternative mRNA isoforms. We support this hypothesis by illustrating that novel, independently regulated transcripts are differentially regulated in the *mdx* mouse, and that conserved myogenic isoforms, in particular utrophin A′ and F, are valid screening targets for our small compound‐mediated utrophin upregulation strategy. Importantly, this approach can be employed in methodologies targeting multiple isoforms or used in synergy with alternative therapeutic strategies to favour an enhanced regenerative response in DMD patients.

## Materials and methods

### RNA ligase‐mediated (RLM) 5′ Rapid Amplification of cDNA Ends (5′RACE)

RLM‐5′RACE (Ambion, Austin, USA) used 5 μg total decapped RNA from human (mix of 1.25 μg each of fetal heart/lung/thymus/skeletal muscle; Agilent, Santa Clara, USA) or mouse (mix of 2.5 μg each pooled whole embryo day 7 and 11; Clontech, Mountain View, USA). Reverse primers within utrophin exon 6 were used for cDNA synthesis (500 ng template) with 5′‐outer adapter RACE primer for primary PCR (95 °C/1 min; 30 cycles 95 °C/45 s, 62 °C/45 s, 72 °C/1 min, final extension 72 °C/5 min). Secondary nested PCR (1/10 input) used 26 cycles with utrophin exon 4 reverse and 5′RACE forward adapter primers. Transcription start sites from PCR products were identified by cloning into a TA‐vector (pCRII‐TOPO; Invitrogen, Carlsbad, USA) and analysing *n* = 60 individual colonies per species. Confirmation of novel utrophin transcripts used isoform‐specific PCR (30 cycles, conditions as above). All PCR products were fully sequenced on both strands.

### Quantitative/semiquantitative reverse transcription PCR (q/sqRT‐PCR)

SYBR Green quantitative RT‐PCR (qRT‐PCR) used 1/20 cDNA template (1 μg total RNA input) synthesised from ≥ 2 different reverse primers within utrophin exons 4–6 (Table S1–S2) with 1/20 used in qRT‐PCR. All reactions used Sensimix; Bioline, Ltd., Canton, MA, USA and Rotor‐Gene 3000 (Corbett Life Science, Crawley, UK) with the exception of studies involving individual hindlimb muscle (Fig. S9; StepOne Real‐Time system and Fast SYBR Green Master Mix; Thermo Fisher, Cramlington, UK). Values were obtained by comparison to primer set standard curves serially diluted 1 : 10 to adjusted length^−1^ (~ 10 ng) and standardised to 28 s rRNA values from diluted cDNA template. Semiquantitative PCR (sqRT‐PCR) for the human adult tissue RNA panel (Zyagen, CA, USA) used 32 cycles of 95 °C/45 s, 62 °C/45 s, 72 °C/1 min, final extension 72 °C/5 min and quantified using ImageQuant (Amersham Biosciences, Piscataway, USA).

### Cell culture and tissue samples

Mouse C2C12 skeletal myoblast 23, 24 and C3H/10T1/2 (C3H) pluripotent stem 25 cells were maintained in DMEM+10% FBS, 37 °C/5% CO_2_. (C2C12: +2% horse serum (HS), C3H: +1 ng·mL^−1^ recombinant human TGF‐β1). H2K cells 26 were cultured in DMEM+20Uml^−1^ γ‐interferon, 2% chick embryo extract, 20% FBS at 33 °C/5% CO_2_ and differentiated in DMEM+5% HS, 39 °C/5% CO_2_ for 6 days. Human immortalised myoblasts 27 were cultured in F10 + 20% FBS at 37 °C/5% CO_2_ and differentiated in 5% HS for 6 days. Cells were differentiated/harvested at 70–75% confluency. Male C57BL/10 wild‐type and *mdx* 10, 11 tissue was dissected, homogenised (Precellys24; Bertin, Montigny‐le‐Bretonneux, France) and RNA isolated (Trizol; Invitrogen, Carlsbad, USA) according to manufacturers’ instructions. Pellets from clonal mesoangioblast (MAB) cell lines D16 and D351 derived from the dorsal aorta of C57/BL6 9.5 dpc embryos 28, isolated *mdx* embryonic aortic/postnatal skeletal muscle MABs and isolated DMD skeletal muscle MABs were a kind gift from G.Cossu and R.Tonlorenzi (San Raffaele Scientific Institute, Milan). Commercial sources of RNA are as follows: (a) mouse embryo; Clontech, Mountain View, USA and (b) human embryo and adult tissue panel; Agilent Santa Clara, USA.

### Luciferase constructs and reporter assays

The interexon region between mouse (640 bp) and human (728 bp) utrophin exons 1A and 1A′ (A^ie^) were cloned in both orientations into the *HindIII* site of pGL4.10[*luc2*] (Promega, Madison WI, USA) and column‐purified (Qiagen GmbH, Hilden, Germany). Mutagenesis of the mouse A^ie^ E‐Box (cagctg to ctgcgg) was performed as per 29. C2C12 cells were seeded (7.5 × 10^4^ myoblast/3 × 10^5^ myotube) and transfected with 0.5 μg construct/20 ng pRL‐TK control plasmid (Promega, Madison, USA) per 12 well. Luciferase activity was measured after 48 h on *n* = 8 separately transfected wells as previously described 30.

### Electrophoretic mobility shift assay (EMSA)

EMSA was performed as outlined in 30 with 8 μg differentiation day 3 C2C12 nuclear extract or 10 μg human over‐expression/control HEK293T lysate (Origene, Rockville, USA). Assays employed 100‐fold unlabelled probe (cold competitor) or 1 μg α‐myogenin (F5D; sc‐2027; Santa Cruz Biotechnologies, Santa Cruz, USA)/rabbit IgG added 30 min on ice prior to probe addition.

### Antibody production and purification

F‐utrophin antibody (‘utroF’) was raised in two pathogen‐free rabbits (denoted 74 and 75) by immunization with synthetic peptide to the unique mouse N terminus (MEETSVATDGASRES) coupled to keyhole limpet hemocyanin carrier protein *via* an additional C‐terminal cysteine (Eurogentec, Southhampton, UK). Quality control during manufacture indicated utroF 74 and 75 antisera did not illustrate reactivity to preimmune serum and were functional in a peptide‐based ELISA assay (Eurogentec, Southhampton, UK). Antiserum immunoglobulin was precipitated by dissolving in 14%(w/v) anhydrous Na_2_SO_4_ (Sigma Aldrich, Poole, UK) at 37 °C/40 min, pelleted 2500 *×* ***g*** for 15 min, dissolved in ½(v/v) H_2_0 and re‐precipitated with 7% (w/v) anhydrous Na_2_SO_4_. Redissolved pellets (1xPBS) were affinity purified using immunising peptide immobilised on SulfoLink (Thermo Fisher, Cramlington, UK) as previously described 17.

### Immunoblotting

Specificity of utroF was assessed by dot‐blots of (a) serially diluted immunising peptide (2.0–0.02 μg) probed with utroA, B and F antibodies (1 : 200, 1 : 50 and 1 : 50 dilution in TBST+5% BSA respectively (Fig. S8c; left) (b) nonreactivity of utroF purified antiserum to utroB immunising peptide (Fig. S8c; right). All blots contained 20 μg total protein from 6‐week *mdx tibialis anterior* (TA) muscle as a positive control. Analysis of utroF control/*mdx* 2‐week diaphragm and 6‐week TA (Fig. S8d) using utroF were performed under identical conditions and imaged using ImageQuant LAS 4000 (GE Healthcare Life Sciences, Little Chalfont, UK).

### Immunofluorescence

Tissue sectioning and immunofluorescence (IF) was performed as described 31. Polyclonal antibodies/dilutions are as follows: NCAM (C‐20; 1 : 200), PCAM (MEC13.3; 1 : 500, Santa Cruz Biotechnology, Santa Cruz, USA), NG2 and aSMA (ab101679; 1 : 500 and ab21027; 1 : 250, Abcam, Cambridge, UK), utroA (1 : 2000) and purified utroF (1 : 100) in 3% BSA (Sigma Aldrich, St Louis, USA). Secondary donkey antibodies used were: α‐rabbit Alexa 488 (1 : 2000), α‐rat Alexa 546 (1 : 1000, Molecular Probes, Leiden, Netherlands and α‐goat Alexa Cy3 (Thermo Fisher Scientific, Cramlington, UK; 1 : 1000). TA sections from 6‐week *mdx* mice were stained (300 mm DAPI; Sigma Aldrich, St Louis, USA), mounted (Hydromount; National Diagnostics, Hessle, UK) and imaged using Olympus FV1000 inverted Fluoview confocal microscopy/FV10‐ASW software (Olympus Corp, Center Valley, USA). Confocal IF was also used to determine UtroF specificity using 6‐week *mdx* and *dko* TA sections (Fig. S8) and use of secondary antibody‐only controls. Analysis of specific skeletal muscle sections outlined in Fig. S9 used identical conditions as above, with α‐rabbit Alexa 488 (1 : 2000) and imaged by upright fluorescence microscopy (Axioplan 2 Microscope System; Carl Zeiss, Germany).

### Sample numbers and statistical analysis

**Figure 2:**
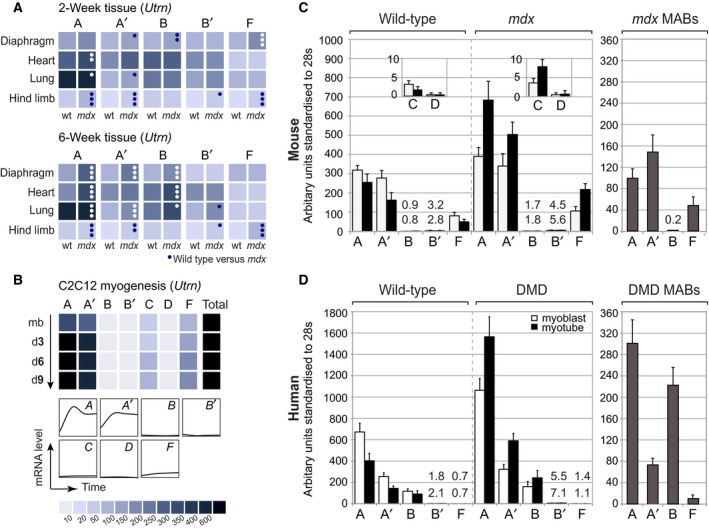
Utrophin isoforms exhibit species‐ and isoform‐specific transcript levels. (A) The postnatal mouse response to dystrophin‐deficiency is tissue and isoform specific. Quantitative RT‐PCR (qRT‐PCR) analysis of tissue samples sourced from control (wt) C57BL/10 and *mdx* C57BL/10 mouse tissue at the 2‐week precrisis period (upper panel) and during the degeneration‐regeneration period in *mdx* during which total *Utrn* levels are elevated (6‐weeks; lower panel). Values for wt are at the left and for *mdx* at the right as denoted below each pair of columns. *Utrn*‐C/D mRNAs were greatly lowered in comparison to other isoforms (Table S2). *P* values represent differences between wild‐type and *mdx* tissues at the same time point (*P* < 0.0001••• *P* < 0.001••, *P* < 0.05•, 99% cl). qRT‐PCR analysis of *Utrn*‐A,‐A′ and ‐F levels in specific hindlimb muscles (*tibilialis anterior*, quadricep and soleus) compared to diaphragm (Fig. 2A) is provided in Fig. S9. (B) Selective upregulation of utrophin isoforms during C2C12 myogenesis. qRT‐PCR analysis of endogenous *Utrn* mRNA levels in myoblasts (myob) and differentiating (diff) myotubes sampled at days 3, 6 and 9, with corresponding mRNA profiles illustrated below. For (A and B), values are represented as a colour scale under (B), standardised to 28s cDNA for each time‐point. Numerical values obtained for all sample sets and additional statistical data are provided in Table S2. (C–D) Species‐specific utrophin mRNA myogenic profiles are mirrored in skeletal muscle mesoangioblasts (MABs). qRT‐PCR of utrophin mRNA isoform levels in wild‐type, *mdx*/DMD myogenic cell lines (left panel; white columns, myoblast, black columns, myotubes as outlined in the legend) and in dystrophin‐deficient skeletal muscle mesoangioblasts (MAB, grey columns, right panel). Values too low to be visualised by scale are provided numerically (upper value; myoblast, lower value; myotube). Numerical values for (A–B) sample sets are provided in Table S2.

sqRT‐PCR (human tissue panel) was performed *n* = 3 per isoform using individually synthesised cDNA template mixes, quantified on separate agarose gels. All qRT‐PCR reactions were conducted in triplicate, numbers as follows. C2C12 myogenesis and C3H studies were performed using cDNA synthesized from up to 4 or 6 wells, respectively (nonpooled), with standard deviation calculated if *n* ≥ 3 separate cDNA preparations used. For prepooled samples (aorta‐/skeletal muscle‐derived *mdx* mesoangioblasts, D16 and D351 clonal cell lines, mouse total embryo, human embryonic tissue), three separate cDNA preparations were performed in triplicate. Data presented in colour scale (Figs 1B–C, 2A–B) are presented in numerical form in Table S1–S2. Postnatal mouse tissue samples were extracted from *n* ≥ 3 subjects; two tailed *t*‐tests and *P*‐values on data in Fig. 2A were conducted using Prism7 (GraphPad, La Jolla, USA) and listed in Table S2.

### Online database resources

Sequence alignments were performed using NCBI BLAST (https://blast.ncbi.nlm.nih.gov/32). Phylogenetic analysis of utrophin N‐terminal peptide sequences used Clusal Omega 33. Next‐generation sequencing data from the University of Santa Cruz California (UCSC) genome browser/Encyclopedia of DNA Elements (ENCODE) aligned to mouse (NCBI37/mm9 assembly) and human (NCBI37/hg19) were accessed *via* http://genome-euro.ucsc.edu/, with data sources as follows: (a) mouse; DNaseI hypersensitivity site (HS) signals: University of Washington, C2C12 RNA‐/ChIP‐seq data: Caltech (b) human; RNA‐seq: Caltech, DNAseI HS raw/density signal: University of Washington and Duke University respectively; histone markers: Broad Institute 34, 35.

### Animal use and ethics statement

The source and use of animal tissue was performed under strict compliance with required standards outlined in the Animals (Scientific Procedures) Act 1986, revised 2012 (ASPA) and European Directive 63/2010 relating to the protection of animals used for scientific purposes.

## Results

### Transcriptional complexity of mouse and human utrophin loci revealed by 5′RACE and genome analysis

As the genomic location and splicing configuration of utrophin A and B are conserved between mouse (Utrn) and human (UTRN) gene loci 13, 14, we hypothesised unidentified novel regulatory transcripts/elements within the ~ 100 kb 5′ region spanning exons 1A‐3 may contribute towards the *mdx*/DMD genotype‐phenotype disparity. Novel exons were identified by 5′RACE using embryonic tissue, when utrophin expression peaks 8. PCR products were cloned and sequenced on both strands to determine transcription start sites. The presence of novel exons were then authenticated using isoform‐specific RT‐PCR, sequenced (Fig. S1–S4) and prefixed according to established nomenclature. Prime symbols (′) were allocated to exons that share sequences previously thought unique to previously identified full‐length utrophin A‐ and B‐mRNAs (A′ and B′ respectively, Fig. 1A).

Utrophin exon 1A′ lies in a conserved region ~ 550 bp 3′ to 1A and gives rise to *Utrn*A′ mRNA (Fig. S1a) *via* cosplicing to translated exon 2A. Thus, any protein product arising from utrophin 1A′ would be indistinguishable from utrophin 1A using mouse 17 and human 15 A‐utrophin specific N‐terminal antibodies. Conversely, utrophin B′ isoforms share a common first exon (1B), but divergent splicing results in distinct open reading frames that retain actin binding potential (Fig. S2). Three novel first exons splicing to exon 3 (1C,D and F) were also identified (Fig. 1A; Fig. S3–S4). As mouse exon 1C is untranslated, potential use of an in‐frame methionine in exon 4 would give rise to a protein indistinguishable using antibody‐based approaches, whereas murine exon 1D contains two potential initiating methionines (Fig. S3). Human *Utrn*‐C and *Utrn*‐D paralogues were not detected, indicating these mRNAs are either (i) mouse‐specific or (ii) absent in either the source tissue selected/within the conserved region selected for *UTRN*‐specific 5′RACE. In contrast, exon 1F was of particular interest; similar to exons 2A and 1B 17, *Utrn‐*/*UTRN*‐F mRNAs contain open‐reading frames and have the capacity to encode unique N‐terminal protein isoforms (Fig. S4). Identification of these novel mRNAs highlights the complexity of *Utrn*/*UTRN* loci, which differ in their splicing and transcriptional attributes.

### Utrophin isoforms are independently regulated in embryonic and adult tissues

With the exception of *Utrn*‐A′, developmental profiling indicates mouse *Utrn* transcription peaks at embryonic day 11 (Fig. 1B, Table S1) with subsequent decline concomitant with the onset of dystrophin transcription in smooth muscle (e11) and organs such as lung (e12) and brain (e13) 36, 37. During early embryogenesis (e7–11), utrophin 2A‐ and 1B‐containing transcripts initially represent major isoforms, but become progressively less apparent when *Utrn* levels are lowest and the overall contribution from alternative mRNAs becomes more pronounced (e15–17; Fig. 1B). As total *UTRN* tissue patterning is similar to that of *Utrn*, we analysed the transcriptional attributes of human paralogues in embryonic and human tissue panels using qRT‐PCR and sq‐RT‐PCR respectively (Fig. 1C, Fig. S5a, Table S1). Comparison of mRNAs that comprise total utrophin A (2A‐:*UTRN*‐A/A′) or B (*UTRN*‐B/B′) indicate that their individual mRNA transcripts have similar isoform‐specific, but overall distinctive, expression profiles, a phenomenon previously indistinguishable by prior studies 13, 14, 17, 38. Although human utrophin isoforms appear independently regulated, common regulatory attributes may also be shared, particularly with *UTRN*‐B/B′ where the main utrophin B′ mRNA start site lies within exon 1B (Fig. S2). Conversely, *UTRN*‐F is particularly distinctive in its tissue specificity, with abundant distribution in mesenchymal lymphatic tissue and highly vascularised organs (Fig. 1C). The latter observation is solidified using UCSC ENCODE DNaseI‐seq data 34, where human exon 1F resides within an open chromatin region in not only embryonic stem cells, but in adult tissues; notably epithelial‐derived cell lines (Fig. S6). Extending analysis to corresponding murine A and F utrophin genomic regions, DNAseI‐seq data (Fig. S6–S7) indicates utrophin exons 1A,A′ and F all reside in open chromatin regions across a broad variety of embryonic and adult tissues.

### Postnatal Utrn mRNA variability in wild‐type and *mdx* tissue

We also wished to establish whether mouse utrophin transcript levels differ between age‐matched wild‐type and *mdx* tissue and thus performed isoform‐specific qRT‐PCR using tissue sourced at the preweaning stage (2 weeks) to determine transcript levels prior to increased activity and onset of visible muscle pathology. These data were directly compared to identical tissues isolated from 6‐week old mice; after the primary ‘wave’ of muscle necrosis (3–4 weeks), when active regeneration, functional restoration and *Utrn* re‐expression occurs 39, 40(Fig. 2A). Postnatal *Utrn* is most abundant in lung 38, 41, 42 total *Utrn*‐A and *Utrn*‐B mRNA levels agree with prior studies 38; and low levels of *Utrn‐C, D* and F (Fig. 2A, Table S2) are comparable with wild‐type mRNA profiles. We also assessed heart, given that cardiomyopathy represents a leading cause of DMD patient death 43. *Utrn*‐A and ‐B heart mRNAs are upregulated in *mdx* (1.7‐fold), with total 1B‐/2A‐containing mRNA profiles similar to prior studies 14, 17, 38. Other isoforms show comparable wild‐type/*mdx* profiles that remain stable or decrease with age (Fig. 2A, Table S2). However, as *Utrn* overexpression in diaphragm ameliorates cardiac symptoms 44, the role of individual isoforms in perpetuating milder *mdx* cardiomyopathy was not pursued.

In diaphragm, the sole *mdx* tissue with severe DMD pathophysiology (45), utrophin A transcripts are markedly elevated in *mdx* at 6‐weeks (*Utrn*‐A; 2.9‐fold and *Utrn*‐A′; 3.2‐fold, Fig. 2A, Table S2), mirrored to a lesser extent by *Utrn*‐B (1.8‐fold). Conversely, *Utrn*‐B′ and ‐F upregulation in *mdx* at the 2 week stage is not retained. Surprisingly, elevated *Utrn*‐F hindlimb levels in *mdx* at the preweaning stage (2.3‐fold) almost doubles at 6‐weeks (4.2‐fold) representing a direct contrast to the concurrent, lower and nonsustained profile in diaphragm (Fig. 2A). As *Utrn*‐A′/F upregulation may be linked to postnatal hindlimb regeneration in *mdx* 19, 20, we wished to determine the myogenic capacity of *Utrn* isoforms for comparison with their human paralogues.

### Myogenic upregulation of endogenous mouse utrophin mRNA *in vitro*

Links between *Utrn* and efficient postnatal *mdx* regeneration are established during reversion to fetal myogenic programming and muscle precursor activation 19, 20, 39, 46. We therefore initially documented levels of endogenous *Utrn* mRNAs during myogenic differentiation in C2C12 cells 23, 24 and show *Utrn*‐A (2.5‐fold) and ‐A′ (2.2‐fold; Fig. 2B) contribute to the established 2‐fold myogenic upregulation of 2A‐containing mRNAs 30, 38, 47. Furthermore, *Utrn*‐C (1.4‐fold) and *Utrn*‐F (1.7‐fold) mRNAs increase during myogenesis, whereas other isoform mRNAs were barely detectable. After consideration of the tissue and myogenic properties of novel transcripts, further characterization of murine utrophin isoforms B′/C/D were not pursued due to the absence of (a) a human paralogue (b) an efficient myogenic response and (c) overall contribution to utrophin transcript levels in multiple tissues.

### Utrophin isoforms exhibit species variability during myogenic differentiation

Although C2C12 cells represent an established myogenic model 23, 24, they do not reflect the *in vivo* transcriptional decline of utrophin during skeletal muscle development 39, 48 nor represent the absence of dystrophin. We therefore compared the transcriptional response of utrophin under wild‐type and dystrophin‐deficient conditions using immortalised cell lines from H‐2K^b^‐tsA58 mice (H2K 26 Fig. 2C) and human skeletal muscle (27 Fig. 2D). Although both systems recapitulate *in vivo Utrn*/*UTRN* myogenic profiles, two marked differences between species exist in conferring this response. The first is the ratio of 2A‐containing mRNAs, where *Utrn*‐A′ mRNAs are more abundant in H2K‐*mdx* than *UTRN*‐A′ in dystrophin‐deficient hDMD cells. Secondly, a reciprocal relationship exists where elevated *Utrn*‐F and low *Utrn*‐B mRNA levels are reversed in their human paralogues. The impeded myogenic/dystrophic response of *UTRN*‐A′/‐F and expression of *UTRN*‐B over human *UTRN*‐F may be linked to differential myogenic signalling involving, in part, E‐Box‐mediated mechanisms.

### The molecular basis of Utrn‐A**′ and Utrn‐F myogenic induction**

**Figure 3:**
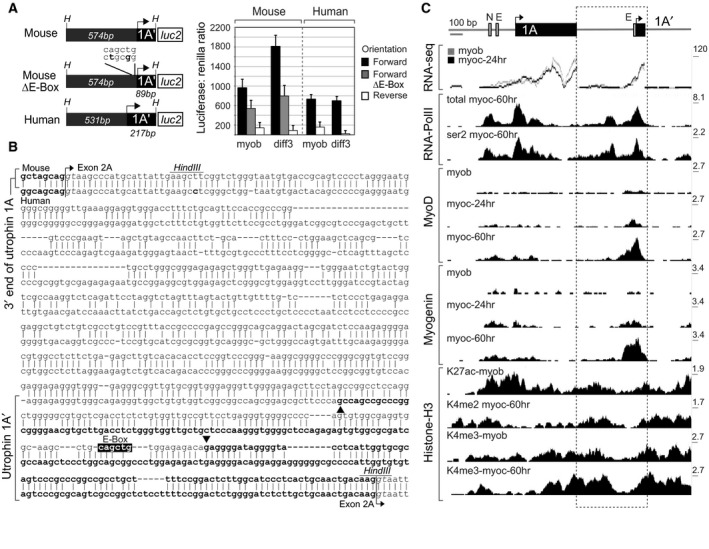
The utrophin interexonic region (A^ie^) facilitates myogenic induction of *Utrn*‐A′. (A) The mouse and human utrophin A^ie^ illustrate the capacity to function as A′ promoter elements. Comparison between transfected C2C12 myoblasts (myob) and 3‐day differentiated myotubes (diff3) lysates indicate myogenic upregulation of *Utrn*‐A′ (a) compromised in the mouse E‐Box mutant (∆E‐Box; grey columns) and (b) absent in the human equivalent. Luciferase constructs are detailed on the left and results represented in grey‐scale per graph legend (right). Error bars; SD, samples obtained from *n* = 8 separately transfected wells/time point. (B) Mouse (upper) and human (lower) sequence alignment spanning the 3′ end of Utrophin 1A to the splice donor site of Utrophin 1A′. Location of murine E‐Box (black), transcription start sites of *Utrn*‐/*UTRN*‐A′ (black arrows; transcribed sequence in bold) and *HindIII* sites used to clone the interexonic region as denoted. Splice junctions to exon 2A are marked with arrows. (C) Utrophin exon 1A/1A′ genomic region aligned with UCSC ENCODE‐Caltech C2C12 ChIP‐seq and RNA‐seq data 34, 35. Arrows; transcription start sites, untranslated exons (black boxes) and E‐/N‐Box motifs (grey boxes), A^ie^ region aligned to UCSC tracks (dashed box). Scale bar provided. MyoD/myogenin 24/60 h differentiation, and active histone (H3K4me2/3; H3K27ac) marks are as denoted on the left of each track. myob, myoblast; myoc, myocyte; Additional DNAseI‐, RNA‐ and ChIP‐seq data encompassing the mouse and human 1A/1A′ region is provided in Fig. S6.

As the mouse and human utrophin A myogenic response relies on a functional CANNTG E‐Box motif (CAGGTG: 30), we sought to establish if *Utrn*‐A′ transcription is dependent on the *Utrn‐*A promoter, or is regulated by the uncharacterised 1A/1A′ interexonic genomic region (A^ie^) lying between exons 1A/1A′ (Fig. 3 and Fig. S1). Analysis of the A^ie^ region using UCSC ENCODE‐Caltech C2C12 ChIP‐seq data 35 indicates extensive methylation (H3K4me2/3) and acetylation (H3K27ac) histone H3 marks that hallmark active transcription and positively acting regulatory regions (Fig. 3C), particularly in the absence of repressive H3K27me3 histone modifications 49, 50. Combined with additional ChIP‐ and RNA‐seq data, this observation suggests the murine A^ie^ region is (a) recognised by myogenic factors (with particular affinity for myogenin containing complexes) specifically the E‐box motif located proximal to the transcription start site of *Utrn*‐A′ and (b) produces mRNA levels commensurate with *Utrn*‐1A (Fig. 3C). The corresponding human region was also assessed using UCSC ENCODE RNA‐ ChIP‐ and DNAseI‐seq data 34, 35 from wild‐type LHCN‐M2 27 and HSMM immortalised skeletal muscle 51 cell lines (Fig. S6), indicating *UTRN*‐1A′ also resides in a region of open, transcriptionally active chromatin by DNAseI‐ and RNA‐seq, the latter albeit at lower levels than *UTRN*‐1A. Furthermore, as focal myogenic regulatory factor (myoD/myogenin) enrichment over *Utrn*‐A/A′ transcription start sites/E‐Box motifs can be distinguished by intensity and differentiation‐coupled increase, we postulated that the CAGCTG motif (a preferential hexamer for myogenin (myoG) and myoD 52) within the mouse A^ie^ plays an important role in *Utrn*‐A′‐independent myogenic responsivity (Fig. 3B).

Given the location and preliminary characterization of the A^ie^, numerous possibilities therefore exist in terms of its regulatory capability, including acting as an enhancer element, or driving transcription of an antisense transcript to exon 1A. Subsequent assessment of mouse and human utrophin A^ie^ function using luciferase assays (Fig. 3A) indicates that both can function directionally to yield 1A′ mRNAs (Fig. 3B), however, the myogenic response observed for mouse A^ie^ (1.8‐fold increase; compared to 2.1‐fold for *Utrn*‐A′ mRNA; Fig. 3A) is not recapitulated in its human, nor E‐Box mutant counterpart. This observation supports the hypothesis that the murine A^ie^ E‐Box can act as a myogenic element. Furthermore, as human utrophin A′ exhibits endogenous myogenic activity (Fig. 2D), additional genomic elements residing outside the A^ie^ are clearly implicated in regulating this response.

**Figure 4:**
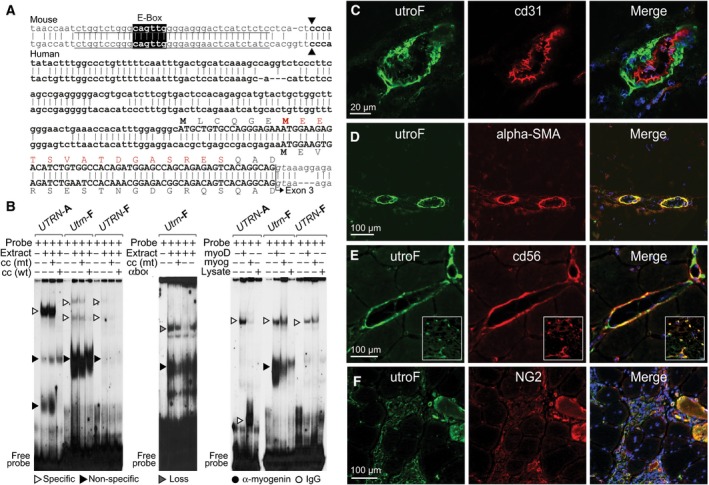
Genomic properties of exon 1F and F‐utrophin distribution in *tibialis anterior* muscle. Overview of utrophin exon 1F, with sequence conservation between mouse (upper) and human (lower), location of E‐Box (black), transcription start sites (black arrows) and putative N‐terminal amino acid sequence as indicated. Lines denote sequences used for EMSA (electrophoretic mobility shift assay) probes; red amino acids denote the peptide sequence used to generate the utrophin murine antibody (utroF) and splice junction to exon 3 is marked with an arrow (canonical intronic splice donor dinucleotide italised). (B: left) *Utrn*/*UTRN*‐F E‐Box motifs indicate distinct protein binding affinity and complex formation from *UTRN*‐A using differentiation day 3 mouse C2C12 nuclear extracts. (B: middle) EMSA analysis using anti‐myogenin (anti‐myoG) antibody and day 3 differentiated cells (‘extract’) with Utrn‐F probe (‘probe’) indicates myoG‐containing complexes. (B: right) Mouse and human utrophin F E‐Box regions have the capacity to bind myoD and myoG as evidenced using human overexpression and control cell lysate. The *UTRN*‐A E‐Box is provided as a positive control. DNA:protein complex type is indicated by arrows (specific; white, nonspecific; black, absence; grey) and addition of antibody by circles (myogenin; black, IgG control; white). ±; addition/absence of labelled components, Probe; radiolabelled double‐stranded probe, extract; 8 μg nuclear extract, cc mt/wt; 100 × excess cold mutant/wild‐type E‐Box competitor, myog/myoD: myoG/myoD overexpression lysate, lysate: control lysate. Panels (C–F): F‐utrophin distribution in *tibialis anterior* (TA) muscle. Dual‐labelled confocal microscopy of 6‐week *mdx* sections using antibodies to F‐utrophin (utroF, left; green), indicated markers (middle, red) and merged images (right; DAPI staining, blue). Interstitial muscle niche colocalization (E–F) and capillary‐like staining (E: inset) noted. Scale bars as indicated. Data on utroF specificity (Fig. S8) and immunofluorescence/qRT‐PCR data profiling mouse utrophin F in specific skeletal muscles (Fig. S9) is provided in Supporting information.

Conversely, *Utrn*‐F transcript levels in C2C12 myoblasts are lower compared to *Utrn‐A/‐A*′ (3.2/2.5‐fold higher respectively) and increase at a later myogenic stage (d9; 1.7‐fold, Fig. 2B). We thus employed a more direct, electrophoretic mobility shift assay (EMSA) using C2C12 nuclear extracts to establish the functional capability of the conserved *Utrn*‐/*UTRN*‐F E‐Box motif (Fig. 4B). Interestingly, mouse and human 1F promoter regions have different affinities for myogenic factor‐containing complexes to *UTRN*‐A 30, including myoD and myoG. It is tempting to speculate the unique *UTRN‐A*: protein complex represents myoD homodimers, as the *Utrn*‐/*UTRN*‐F CAGTTG motif illustrates preference for heteromeric myoG‐E12 and MyoD‐E2A complexes 53. This would account for weakened human *UTRN*‐F probe binding in comparison to mouse (despite identical core hexamers; Fig. 4B) as heterodimerised complexes are particularly reliant on flanking nucleotides for affinity 53.

### F‐utrophin distribution overlaps A‐ and B‐utrophin in mdx skeletal muscle

Similar to 2A‐ and 1B‐containing mRNAs, exon 1F potentially encodes a unique N‐terminal full‐length utrophin protein (Fig. 4, Fig. S4). Given the potential of F‐utrophin to contribute to regeneration/sarcolemmal stabilization of *mdx* skeletal muscle, we sought to identify where resultant protein is localised, particularly as prior studies indicate that total utrophin can be accounted for by A‐utrophin and ‐B only 17. Although not shared by a common utrophin isoform, we confirmed the putative N‐terminal peptide encoded by exon 1F is wholly discrete from A‐utrophin and ‐B using multiple sequence alignment and phylogenetic analysis (33; Fig. 8A) prior to raising a rabbit polyclonal antibody to the predicted N terminus of mouse F‐utrophin (‘utroF’). Antibody specificity was confirmed by numerous means, including dot blots using (a) utroB and utroF immunising peptides (b) control and *mdx* tissue (Fig. S8c) and (c) immunostaining using *tibialis anterior* (TA) sections from dystrophin/utrophin negative *dko* 17, 22 (Fig. S8d).

Dual confocal microscopy of 6‐week *mdx* TA (Fig. 4D–G) localised F‐utrophin to regenerating fibres, perivasculature and the interstitial endomysium. In blood vessels, A‐ and B‐utrophin show discrete localization to the *tunica media* and *tunica intima* respectively 17. Similar to prior results using A‐utrophin antibody 17, F‐utrophin is present in the *tunica media*, evidenced by colocalization with α‐smooth muscle actin, and discrete from the B‐utrophin containing endothelial layer (established using marker PCAM/cd31; Fig. 4D). Incomplete overlap in UtroF/PCAM microvessel costaining indicates that F‐utrophin is present in pericytes embedded within the basement vessel membrane. Furthermore, regional colocalization of UtroF within immature muscle fibres and the interstitial endomysium was observed with neuron‐glial factor‐2 (NG2): staining myogenic pericytes such as mesoangioblasts (MABs: 54, 55) and NCAM, a reliable index of *mdx* regenerating tissue 56, 57, illustrates that F‐utrophin has the capability to participate in the perivascular‐driven myofibre regenerative process. We confirmed our observations by performing additional immunofluoresence on 6‐week TA and other skeletal muscle sections (quadriceps, soleus and diaphragm), including wild‐type controls (Fig. S9b). Interestingly, concurrent qRT‐PCR analysis (Fig. S9a) demonstrated that *Utrn*‐F reflects previously determined total *Utrn* transcript levels 58 and utrophin A in particular 59, 60 by being higher in wild‐type slow, oxidative muscle (soleus; 3.1‐fold) compared to those predominately consisting of fast fibres (TA). This tissue difference was more marked comparing *mdx* soleus and TA (12.9‐fold, 3.4‐fold and 8.8‐fold for Utrn‐1A, ‐A′ and ‐F respectively). Furthermore, the difference between *mdx* over control *Utrn*‐F mRNA levels illustrated the same trend (3.8‐fold in soleus compared to 1.3‐fold in TA). We thus establish that mouse F‐utrophin not only shares nonsarcolemmal attributes previously attributed solely to A‐ and B‐utrophin, but may be regulated by regenerative mechanisms influenced by oxidative capacity.

### Utrophin mRNAs in perivascular mesenchymal stem cells parallel species‐specific myogenic profiling

Given the localization of F‐utrophin and its species‐specific dystrophic response at the mRNA level appear myogenic, we also wished to determine if this phenomena could be linked to vessel‐derived muscle regeneration. Vascular‐derived mesenchymal precursors potentiate myogenic regeneration in diseased muscle 54, 55. In particular, the mesoangioblast (MAB) pericytic subset can restore dystrophin‐deficient muscle and thus represent an attractive DMD stem cell‐therapy approach (61, for review 62).

As F‐utrophin appears perivascular, we monitored utrophin mRNAs during transforming growth factor β1 (TGF‐β1) induced lineage differentiation of the mouse C3H10T1/2 *in vitro* model (C3H; 25) into smooth muscle 63, 64 (Fig. S5b) and in wild‐type MAB cell lines established from embryonic mouse dorsal aorta (D351 and D16 28; Fig. S5c). Utrophin 2A mRNAs were upregulated during C3H differentiation (~ 2‐fold), particularly at 48 hrs when αSMA expression is highest 63, 64, 65. Commensurate with this, D351 *Utrn*‐A/A′ mRNA elevation over D16 MABs (Fig. S5c) matches total *Utrn* array data in pericytes 65 and supports that 2A‐mRNAs positively respond to smooth‐muscle signalling, as D351 are further progressed down this lineage than D16 28. Conversely, *Utrn*‐F appears independent of TGF‐β1‐related signalling and is preferentially expressed in cells retaining pluripotency (Fig. S5b‐c).

Links between lineage preference and dystrophic *Utrn* upregulation ability were then assessed using wild‐type/*mdx* aortic MABs. Direct comparison using cells representing different stages of pluripotency proved complex, as *Utrn*‐A‐/F transcripts were elevated; however, unlike *Utrn*‐A, high D351 *Utrn*‐A′ levels did not further increase in *mdx* MABs isolated directly from aorta (Fig. S5c). We thus specifically compared *mdx* and DMD MABs sourced from skeletal muscle, which not only recapitulated differences between *UTRN*/*Utrn* transcript levels and ratios observed between dystrophin‐deficient myoblasts, but were more pronounced, particularly in DMD MABs (Fig. 2C,D). This furthers our hypothesis that mouse and human loci have evolved separate, possibly myogenic, strategies in response to dystrophin deficiency.

## Discussion

Utrophin is regarded as a regeneration‐associated protein, as its expression is linked to effective myogenic re‐programming that enables functional restoration of *mdx* skeletal muscle. In the *mdx* mouse model, postnatal *Utrn* re‐expression is hallmarked by upregulation within the sub‐sarcolemma and vascular perimysium, a compensatory mechanism that correlates with DMD disease severity but without the protective effects observed in *mdx* mice (reviewed in 66, 67). As dystrophin‐utrophin (*dko)* knockout mice are a more accurate reflection of the DMD phenotype 21, 22, our hypothesis was that the mouse utrophin locus possesses a transcriptional response to dystrophic conditions that differs from its human equivalent. We therefore focused on the isolation of mouse (*Utrn*) and human (*UTRN*) utrophin transcripts with novel 5′ sequences and characterising their transcriptional and species‐specific attributes. Encouragingly, the observed enhanced capability in *mdx* appears to be conferred by differential regulation of *Utrn* isoforms with human paralogues, rather than solely murine‐specific mRNAs.

First, this phenomenon is manifest for utrophin A 13 and B 14 which this study identifies both as two independently regulated mRNAs (A/A′ and B/B′). The 2A‐containing transcripts share myogenic attributes; however, their independency is marked by preferences for *Utrn*‐A′/*UTRN*‐A upregulation during dystrophin‐deficient myogenesis, and within myogenic stem cells such as MABs. Myogenic E‐Box mediated mechanisms within the mouse A^ie^ may elevate levels of extrajunctional *Utrn*‐A′ mRNA that can contribute to sarcolemmal A‐utrophin protein in *mdx*. This phenomena is possibly compromised in DMD patients given the absence of an E‐Box in the human A^ie^ region. Importantly, however, both DMD myoblasts and MABs exhibit the presence of myogenically responsive *UTRN*‐A′ (albeit at lower levels), suggesting elements outside the A^ie^ are likely to confer positive mediation of myogenic signalling or increase post‐transcriptional stability. The role of 1B exon derived isoforms appears more complex. Although *UTRN*‐B′ illustrates similarities in its transcriptional profile with its shared exon 1B counterpart (*UTRN*‐B), the lack of myogenic capability, coupled with nonconserved splicing/putative translation attributes, indicates that an *UTRN*‐B′‐derived strategy is unlikely to be translated to human benefit.

Secondly, we identify a novel transcript, utrophin F, which demonstrates such marked regulatory differences between mouse and humans that we immediately considered its transcriptional control as a primary utrophin candidate in modulating the *mdx* phenotype. Indeed, *Utrn*‐F is the sole non‐2A‐containing transcript upregulated in *mdx* skeletal muscle, during *in vitro* myogenesis and within MABs to levels unparalleled by its DMD paralogue. Links between *Utrn*‐F and enhanced *mdx* regeneration were provided by robust transcriptional upregulation in regeneration‐competent hindlimb (particularly soleus), that is absent in regeneration‐inhibited diaphragm. Using confocal microscopy, we localised F‐utrophin protein with regeneration‐rich regions in *mdx* skeletal muscle, including immature myofibres and the interstitial endomysium, where mesenchymal‐lymphocyte mediated regeneration occurs 68. This region contains multipotent vascular‐derived/mesenchymal precursors with myogenic potential such as pericytes and MABs with increased *Utrn* levels 65, 68 and assist, incorporate or even form, myofibres (reviewed in 55). Our hypothesis that *Utrn*‐F is linked to these events is further bolstered by preferential transcription in mesenchymal cells that retain pluripotency, and upregulation in *mdx* MABs. Critically, as *Utrn*‐F does not appear to be directly regulated *via* TGF‐β1‐inducible pathways, upregulation within the myofibre niche is unlikely to induce fibrosis or inhibit vital regenerative processes such as satellite cell proliferation, myofibre fusion or muscle‐specific gene transcription, a hurdle that currently prevents direct TGF‐β1 targeting strategies from entering the clinic. Isolation of *Utrn*‐/*UTRN*‐F thus allows development of a novel upregulation strategy linked to the vascular‐derived regeneration process to combat nonsarcolemmal DMD pathology.

Dissecting transcriptional and genomic properties of established and novel isoforms alike, we illustrate the utrophin locus displays a considerably more complex pattern of transcriptional regulation and interspecies variation than previously expected. Encouragingly, utrophin A′ and F isoforms exhibiting desirable upregulation traits in mouse (such as the ability to respond to myogenic and regenerative stimuli) have independently regulated human paralogues, providing novel pharmacological targets to supplement promoter‐based small compound approaches focussed on *UTRN*‐A. We thus envisage that a multitargeting utrophin upregulation approach has therapeutic promise in countering sarcolemmal and nonsarcolemmal pathology within DMD skeletal muscle.

## Author contributions

KJP conceptualised and performed experiments, analysed and interpreted data; KED supervised the study, provided experiment advice, and assisted with data interpretation. KJP wrote the manuscript, KED and KJP made manuscript revisions.

## Supporting information


**Fig. S1.** Mouse and human sequence alignment of utrophin exon 1A and 1A′ genomic region.
**Fig. S2.** Mouse and human sequence alignment of utrophin exon 1B and 1B′ genomic region.
**Fig. S3.** Mouse and human sequence alignment of utrophin exon 1C and 1D genomic regions.
**Fig. S4.** Mouse and human sequence alignment of utrophin exon 1F genomic region.
**Fig. S5.** Data on utrophin isoforms to accompany Figs 1 and 2.
**Fig. S6.** Additional information provided by UCSC ENCODE Data.
**Fig. S7.** UCSC DNAseI‐seq information for mouse and human utrophin 1F.
**Fig. S8.** F‐utrophin N‐terminal sequence and specificity of UtroF antibody.
**Fig. S9.** Utrophin F transcript levels and protein distribution in skeletal muscle.
**Table S1.** *Utrn/UTRN* qRT‐PCR and sqRT‐PCR values to accompany Fig. 1.
**Table S2.** *Utrn qRT‐PCR* values and statistics to accompany Fig. 2.
