# Supplementary material for: Alternative utrophin mRNAs contribute to phenotypic differences between dystrophin‐deficient mice and Duchenne muscular dystrophy
Source: FEBS Lett. 2018 May 30;592(11):1856–69. doi: 10.1002/1873-3468.13099 (PMC6032923; doi:10.1002/1873-3468.13099)
Supplement: Supplementary file 1 — Fig. S1. Mouse and human sequence alignment of utrophin exon 1A and 1A′ genomic region. Fig. S2. Mouse and human sequence alignment of utrophin exon 1B and 1B′ genomic region. Fig. S3. Mouse and human sequence alignment of utrophin exon 1C and 1D genomic regions. Fig. S4. Mouse and human sequence alignment of utrophin exon 1F genomic region. Fig. S5. Data on utrophin isoforms to accompany Figs 1 and 2. Fig. S6. Additional information provided by UCSC ENCODE Data. Fig. S7. UCSC DNAseI‐seq information for mouse and human utrophin 1F. Fig. S8. F‐utrophin N‐terminal sequence and specificity of UtroF antibody. Fig. S9. Utrophin F transcript levels and protein distribution in skeletal muscle. Table S1. Utrn/UTRN qRT‐PCR and sqRT‐PCR values to accompany Fig. 1. Table S2. Utrn qRT‐PCR values and statistics to accompany Fig. 2. [file FEB2-592-1856-s001.pdf]

FIGURE S1

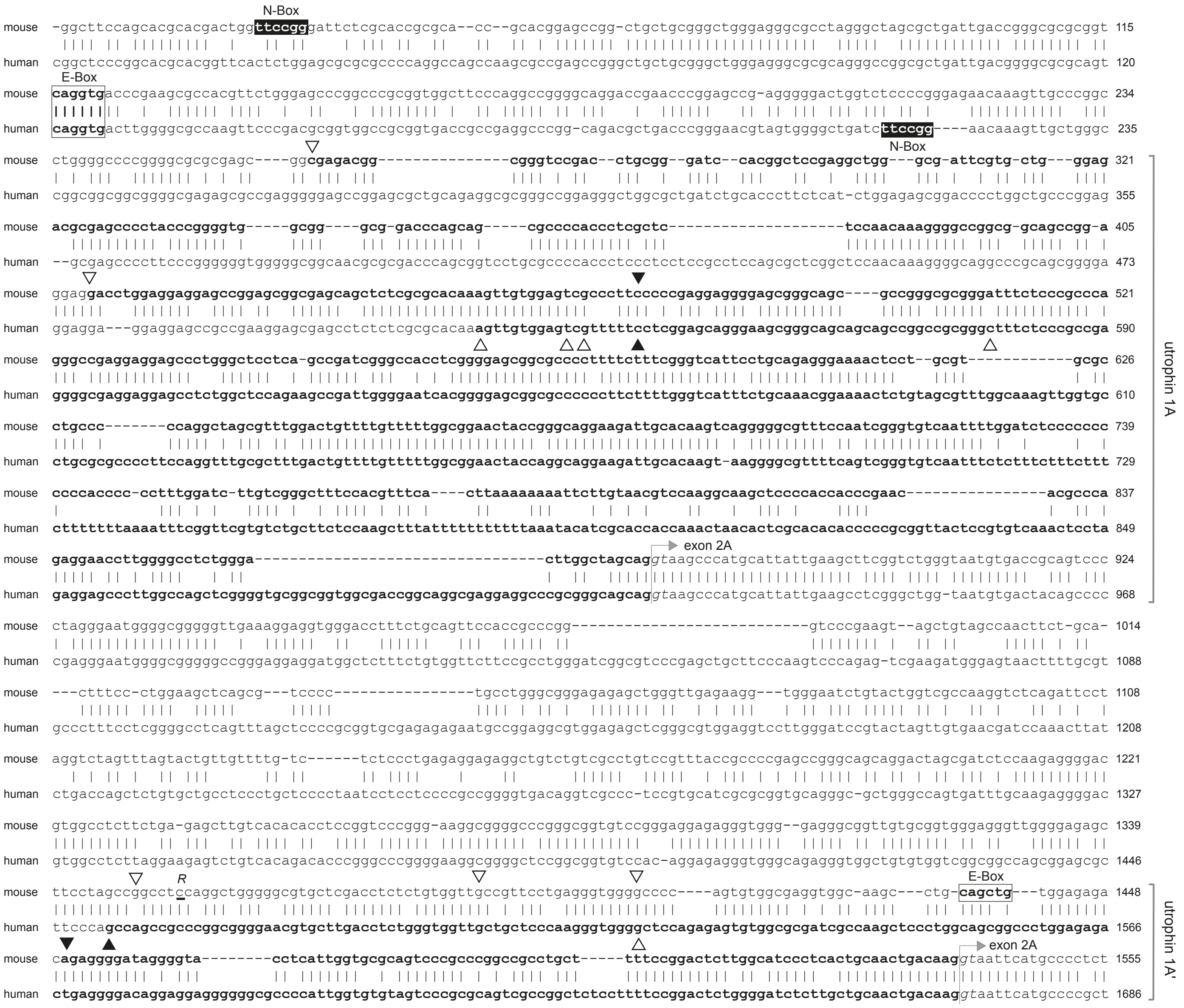

Supplementary Figure 1: Mouse and human sequence alignment of utrophin exon 1A and 1A' genomic region.

Genomic alignment of mouse utrophin exon 1A/1A' (upper; NCBI: NT_039492.7, 5292880-5291331) and corresponding human sequence (lower: NT_025741.15, 8775399-48777182 [32]), including flanking regions. Bold nucleotides span the longest sequence identified by 5'RACE, with synaptic N-Box (black) and myogenic E-Box (black border) motifs denoted. Previously defined major transcription start sites for exon 1A [13], and those determined in this study for exon 1A' are denoted by black arrowheads. Minor transcription start sites for utrophin exons 1A and 1A' are delineated by white arrowheads (for mouse exon 1A, these represent sites identified by GenBank: AK134424.1 and CD561486.1). Italic nucleotides delineate canonical splice donor dinucleotide and bent grey arrows indicate splicing from exons 1A and 1A' to exon 2A.

FIGURE S2

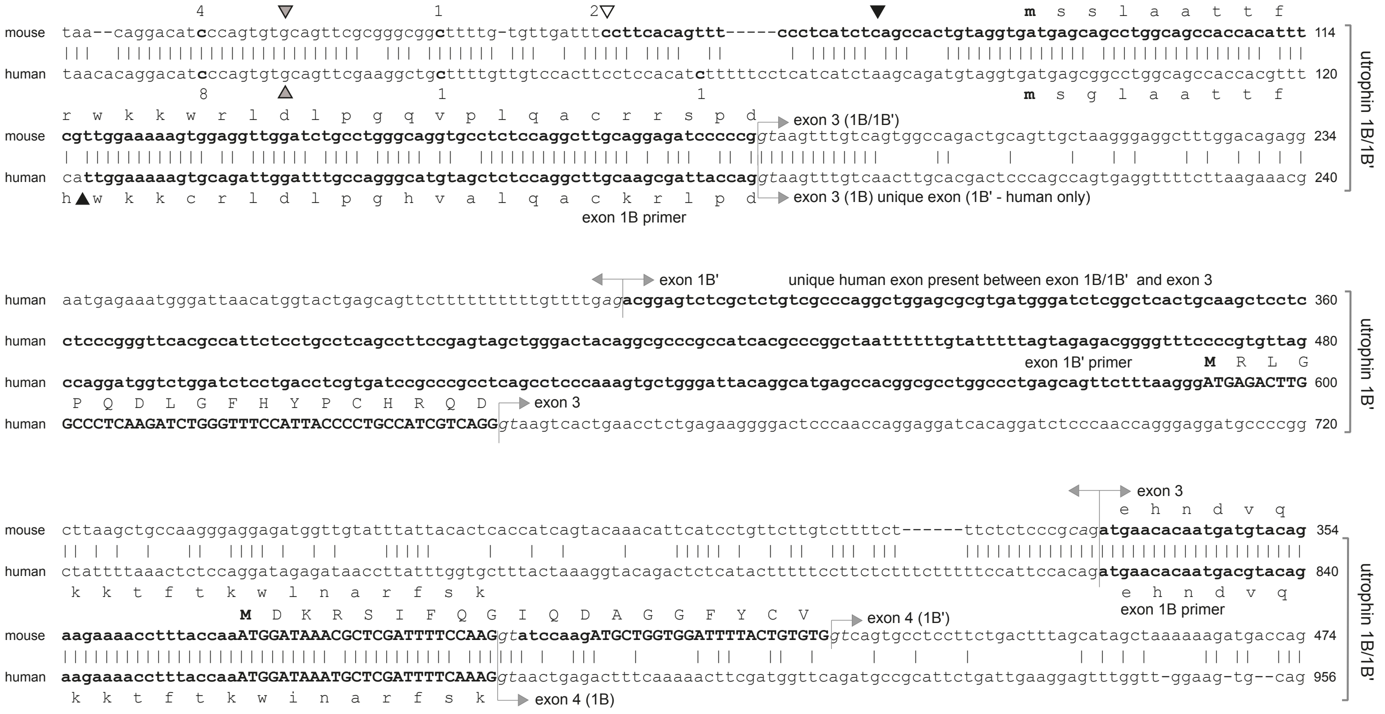

Supplementary Figure 2: Mouse and human sequence alignment of utrophin exon 1B and 1B' genomic region.

Genomic alignment of mouse utrophin 1B / 1B' (upper; NCBI Reference: NT_039492.7; 5236880-5236649; 5188688-5188449) and the corresponding human sequence (lower; NCBI Reference: NT_025741.15; 48834711-48834950; 48839570-48840049; 48893615-48893851 [32]) including flanking regions. Major exon 1B transcription start sites are denoted by grey arrowheads, with minor sites labelled according to number of isolated 5'RACE clones [14]. For exon 1B', bold nucleotides represent the longest sequences identified by 5'RACE, with transcription start sites represented by black (main) and white (minor) arrowheads. The middle sequence represents a unique exon between 1B' and exon 3 which is present in the human *UTRN*-B' mRNA. The mouse *Utrn*-B' sequence has the same splice pattern as 1B to exon 3, where incorporation of extended exon 3 sequence results in an unique open reading frame. Lower and uppercase letters represent translated (1B) or putative unique translated (1B'-containing) sequence respectively. Italic nucleotides the canonical splice donor dinucleotide, with the bent arrow indicating the splice position to the exon specified. The mouse equivalent to human 1B' and the human equivalent to mouse 1B' were not identified using 5'RACE or comparative sequence analysis.

FIGURE S3

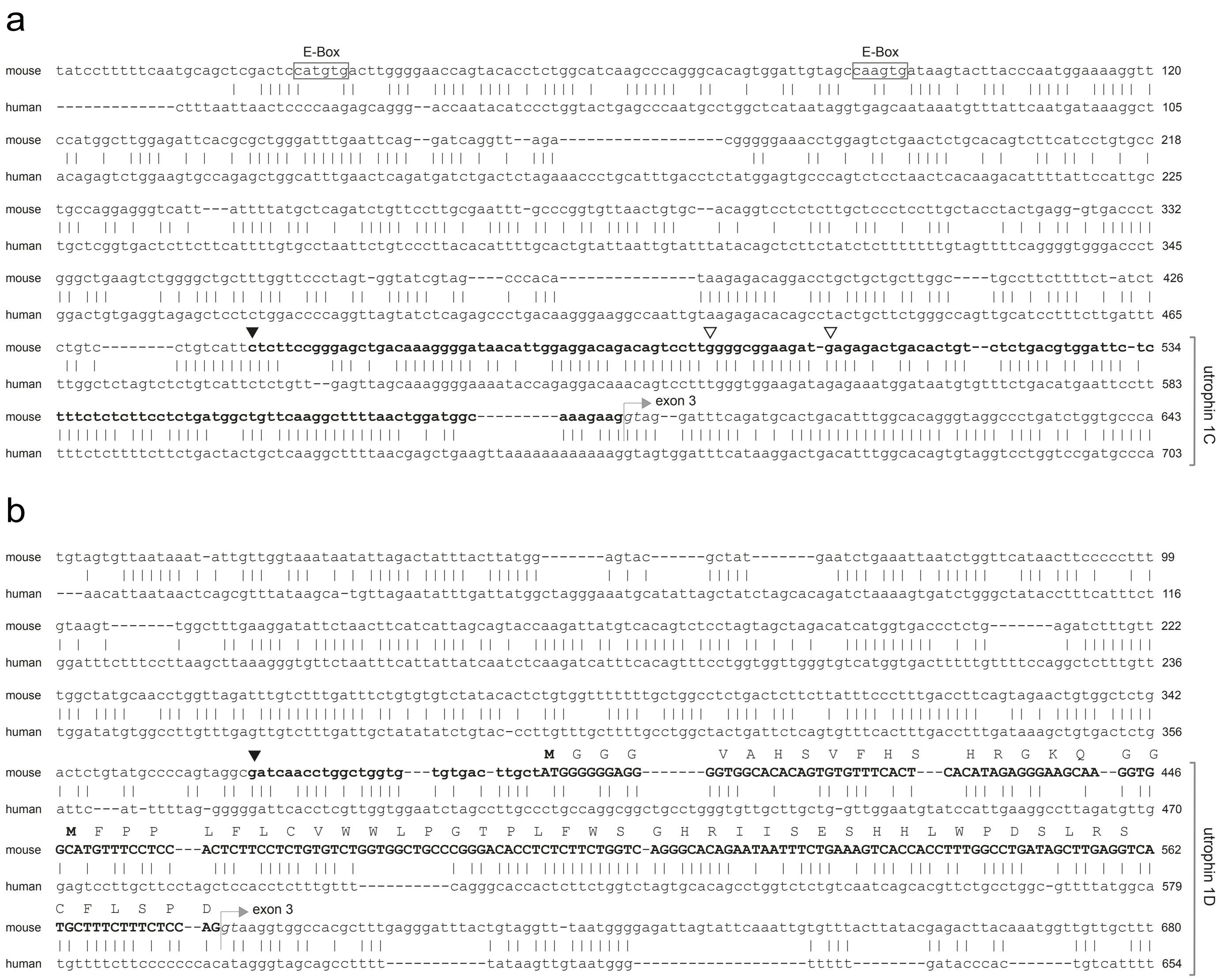

Supplementary Figure 3: Mouse and human sequence alignment of utrophin exon 1C and 1D genomic regions.

Genomic alignment of mouse (A) utrophin 1C and (B) utrophin 1D (upper; 1C: NCBI Reference: NT_039492.7; 5277780; 1D: 5212924-5212263) and corresponding human sequences (lower; 1C: NCBI Reference: NT_025741.15; 48787052-48787754; 1D: 48865931-48866583 [32]), including flanking regions. Bold nucleotides represent the longest transcribed sequence identified by 5'RACE, with black and white arrowheads representing major and minor start sites, respectively. The putative non-conserved myogenic E-Box elements for mouse exon 1C are boxed, and the proposed in-frame translated sequence for mouse 1D is capitalised (potential initiating methionines in bold). Nucleotides in italics delineate the canonical splice donor dinucleotide; bent arrow: splice site to utrophin exon 3. Human transcripts equivalent to *Utrn*-C and *Utrn*-D were not identified using 5'RACE or comparative sequence analysis.

FIGURE S4

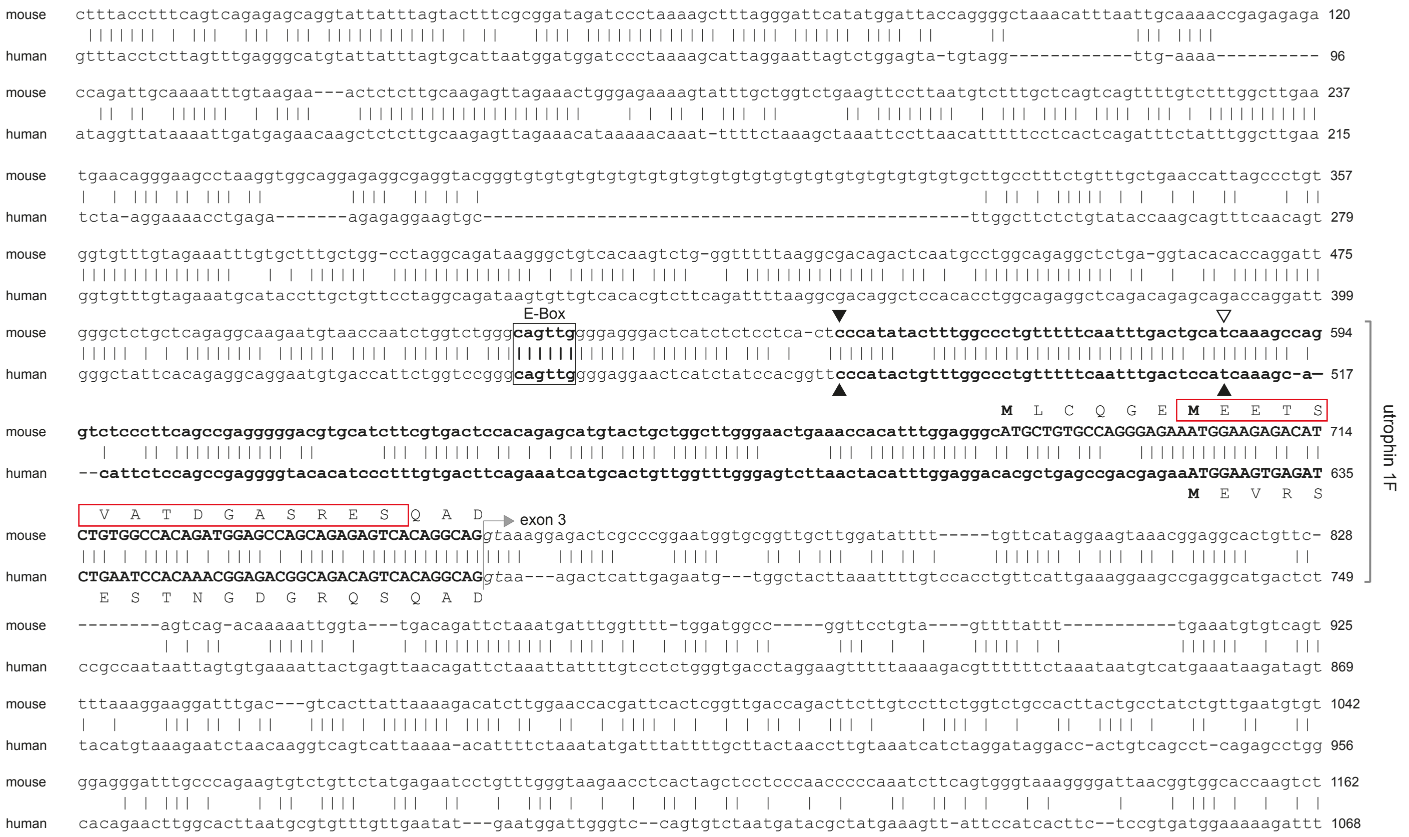

Supplementary Figure 4: Mouse and human sequence alignment of utrophin exon 1F genomic region.

Genomic alignment of utrophin 1F mouse (upper; NCBI Reference: NT_039492.7; 5250776-5249626) and corresponding human sequence (lower; NCBI Reference: NT_025741.15; 48820937-48822035 [32]), including flanking regions. Bold nucleotides represent the longest sequence identified by 5'RACE with black and white arrowheads representing major and minor transcription start sites respectively. The conserved E-Box element characterised in this study is outlined by a box, and the putative in-frame translated sequence is indicated above (potential initiating methionines in bold. The peptide sequence used for UtroF antibody production is outlined by a red box. Nucleotides in italics delineate canonical splice donor dinucleotides; bent arrow: splice to utrophin exon 3.

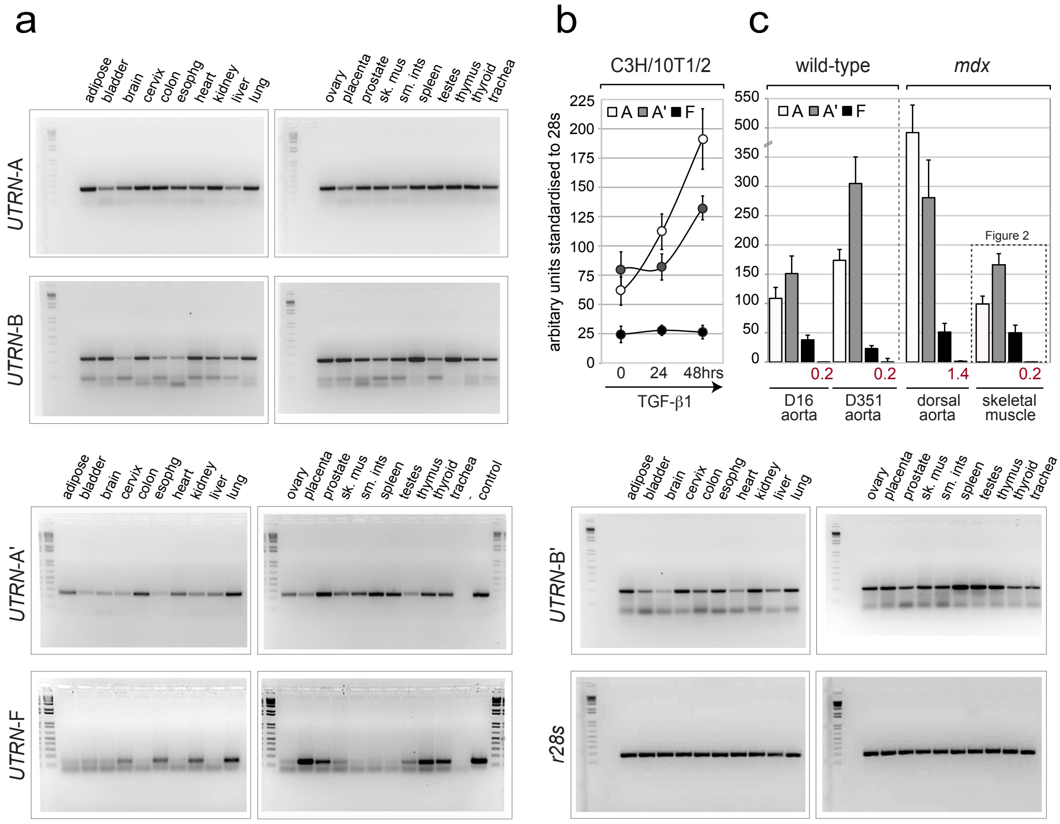

Supplementary Figure 5: Data on utrophin isoforms to accompany **Figures 1** and **2**.

(**a**) Representative full-length sqRT-PCR agarose gels (with 1kb ladder on left) for expression analysis of *UTRN* isoforms outlined in **Figure 1c**. Abbreviations: sk. musc; skeletal muscle, esophg; esophagus, sm. ints; small intestine. (**b**) Transcriptional response of Utrn mRNAs during TGF-β1 mediated smooth muscle differentiation of pluripotent mesenchymal C3H-10T1/2 (C3H) stem cell precursors. Levels of A- and 1F-containing utrophin transcripts were determined using qRT-PCR from total RNA obtained during proliferation (C3H-0) or with TGF-β1 stimulation for 24 (C3H-24) or 48 (C3H-48) hours. (**c**) Isoform-specific mRNA levels in mouse clonal aortic embryo-derived mesoangioblast (MAB) cell lines (D16 and D351 [28]) and cells directly sourced from mdx embryonic aorta. *Utrn*-B levels unable to be visualised by scale are provided numerically in red. Values obtained from skeletal muscle sourced mouse MABs (dotted box; see **Figure 2c**; right panel) are provided for comparison. Sourced sample sets used in sqRT-PCR are outlined in **Table S1**.

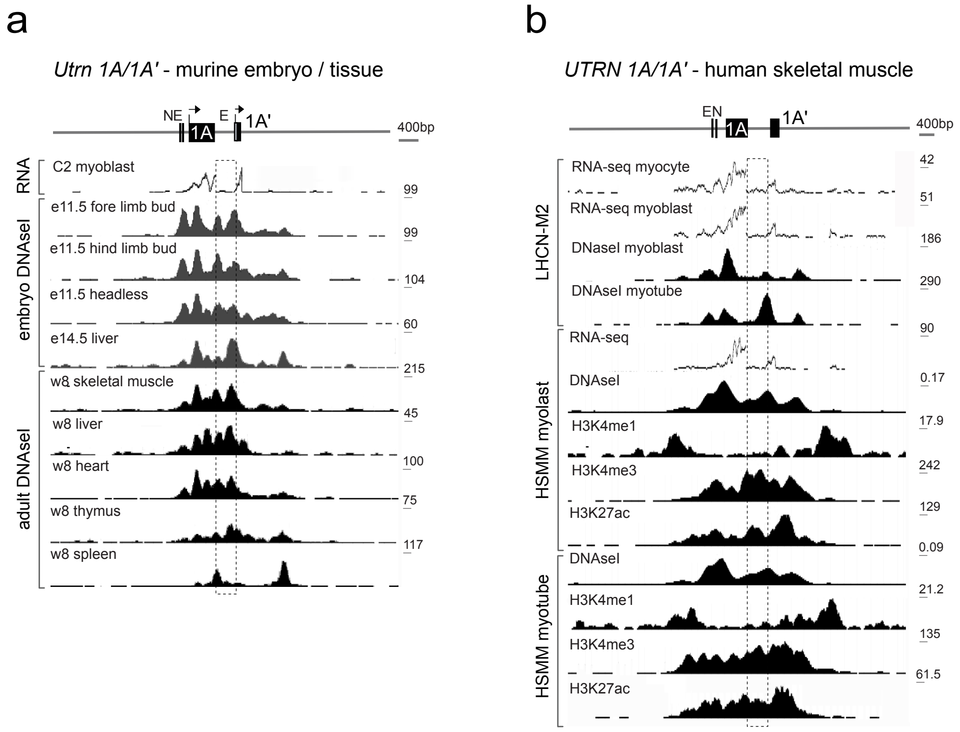

Supplementary Figure 6: Additional information provided by UCSC ENCODE Data.

(**a**) Mouse exon 1A/1A' genomic region aligned with University of California Santa Cruz (UCSC) ENCODE-Caltech embryonic (e: embryonic day) / adult (w: week of life) murine DNAseI hypersensitivity (HS) tracks provided by ENCODE-University of Washington [34, 35] C2C12 (C2) RNA-seq data from ENCODE-Caltech is provided for comparison. (**b**) human exon 1A/1A' genomic region aligned with LHCN-M2 (immortalised pectoralis major) and HSMM (immortalised gestational quadricep) normal human cell line data tracks sourced from UCSC ENCODE-Caltech (RNA-seq), UCSC ENCODE-University of Washington (DNAseI: LHCN-M2), UCSC ENCODE-Duke University (DNAseI: HSMM) and UCSC ENCODE-Broad Institute (histone ChIP-seq) [34]. Active histone marker ChIP-seq tracks (H3K4me3; H3K27ac; H3K4me1) as indicated. For (**a**) and (**b**), numbers represent raw signal intensity (excepting DNAseI HSMM data provided as density signal), arrows; transcription start sites, untranslated exons (black boxes) and E-/N-Box motifs (grey boxes), inter-exonic region aligned to UCSC data (dashed box). Scale bar provided. Additional information is supplied in Materials and Methods.

FIGURE S7

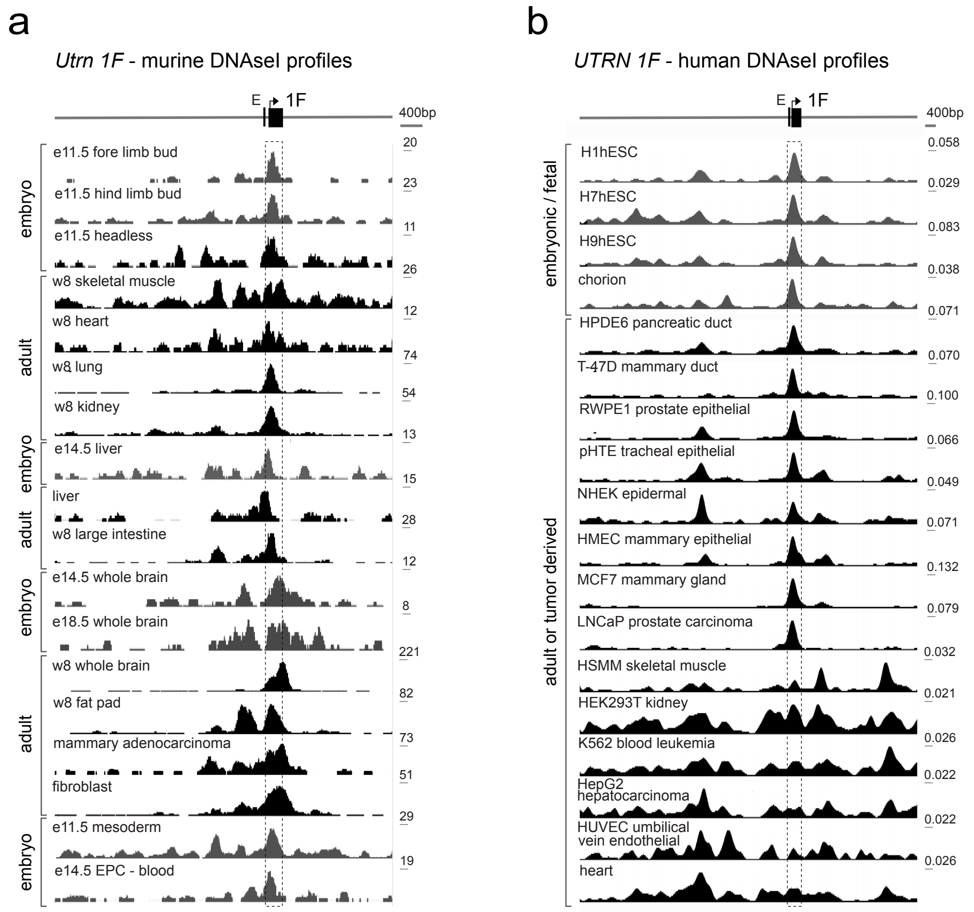

Supplementary Figure 7: UCSC DNAseI-seq information for mouse and human utrophin 1F. Open chromatin (DNAseI hypersensitivity) tracks aligned to mouse (**a**) and human (**b**) utrophin 1F genomic regions sourced from the University of Santa Cruz California (UCSC) genome browser [34, 35]. (a) Exon 1F mouse genomic region aligned with UCSC ENCODE-University of Washington (UW) DNAseI HS signal tracks. Embryo and adult derived as denoted (e: embryonic day, w: week of life). (**b**) Exon 1F human genomic region aligned with UCSC ENCODE-Duke University DNAseI HS Density Signal tracks, cell lines denoted with tissue source. Legend: arrows; transcription start sites, black boxes; untranslated exons, and E-Box / N-Box; “E” and “N” respectively. Regions flanked by a dashed box indicate location of each exon on tracks below. Further information is supplied in Materials and Methods.

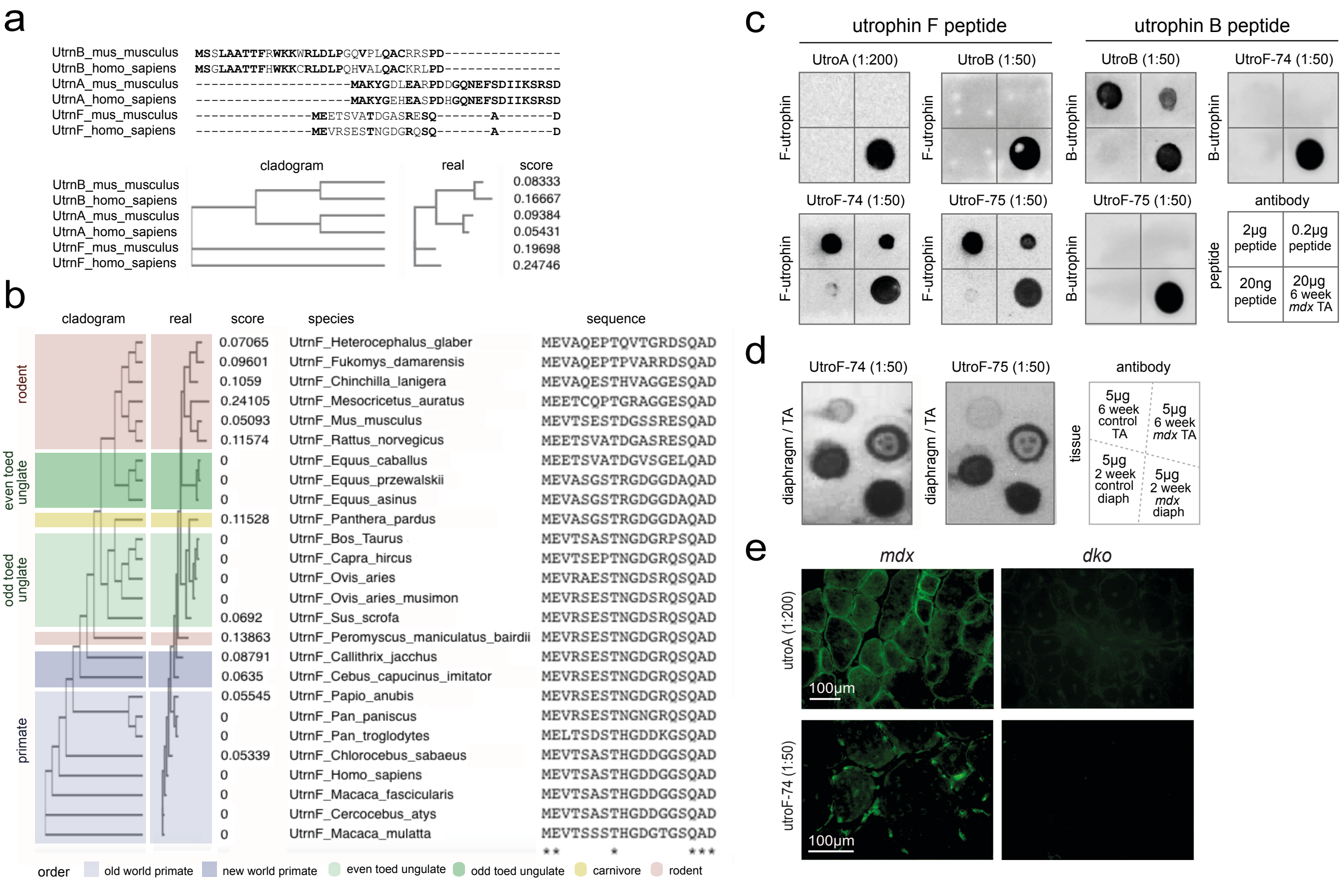

Supplementary Figure 8: F-utrophin N-terminal sequence and specificity of UtroF antibody.

(**a**-**b**) Sequence alignment and evolutionary genetics of the F-utrophin N-terminus indicates divergence from A- and B-utrophin using using NCBI Blastp [32] and Clusal Omega [33]. (**a**) Comparison of mouse and human N-terminus sequences unique to A- (UtrnA), B- (UtrnB) and F-utrophin (UtrnF) with amino acids conserved between mouse and human isoforms in bold. (**b**) Species conservation of the F-utrophin N-terminal sequence, order represented in colour overlay as outlined in the legend. For (**a**-**b**), two seeded phylogenetic representations are shown; "cladogram" reflects the hypothetical relationship between sequences, "real" represents a tree based on evolutionary time and the amount of sequence change (with score referring to branch length from nearest node). (**c**-**e**) Specificity of UtroF rabbit polyclonal purified antisera (dual immunisation denoted as number 74 and 75) raised against the predicted N-terminus of mouse F-utrophin (see Materials and Methods and **Figure S4**). (**c**) Immunising peptide dilution series (utrophin F;left, utrophin B; right) probed with utroA (1:200), utroB (1:50) and antisera from utroF 74 and utroF 75 (1:50) as indicated in the legend, with 20µg total *mdx tibialis anterior* (*mdx* TA) preparation as a positive control. (**d**) Age-matched dot blots of control and *mdx* skeletal muscle using utroF-74 (left) and utroF-75 (right) with control / mdx 6 week TA and control / *mdx* 2 week diaphragm. The amount of tissue used (in µg) is denoted in the legend. (**e**) Purified antiserum 74 (identified as "utroF" in main text) was used to determine specificity by confocal microscopy of 6 week *mdx* and *dko* TA tissue sections (lower panel). Staining with utroA (upper panels) is provided for comparison.

FIGURE S9

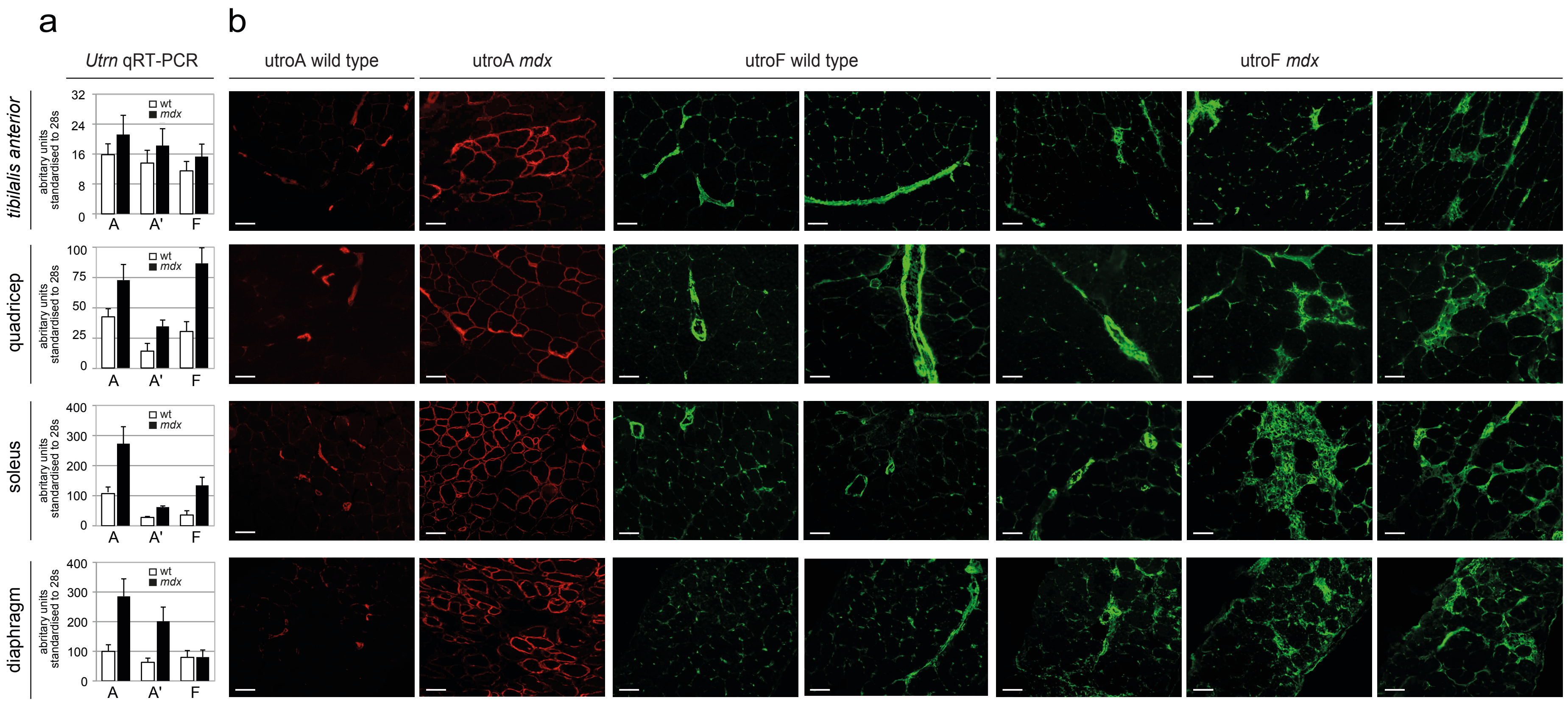

Supplementary Figure 9: Utrophin F transcript levels and protein distribution in skeletal muscle.

Quantitative RT-PCR (qRT-PCR) analysis of *Utrn*-F in individual hindlimb muscles (*tibialis anterior*, quadriceps and soleus) in 6 week control and *mdx* tissue (white and black columns respectively), relative to *Utrn*-A and -A'. Results obtained for diaphragm (see **Figure 2a** and **Table S2**) are provided as a comparison. Values were obtained from n=3 biological replicates for each mouse / tissue type, performed in triplicate using three separately prepared cDNA templates. (**b**) Immunofluoscence (IF) of 6 week control and *mdx* tissue as denoted in (**a**) using utroA (1:200: left panels, red) and utroF (antisera 74, 1:100: right panels, green) antibodies indicate isoform-specific differences in protein localisation and intensity. All images were taken at 20x magnification with identical exposure time per tissue between control and *mdx* sections, scale bar = 100µm. Each representative IF image was sourced from individual mice (e.g., for utroF each tissue denoted: n=2 for wild type and n=3 for *mdx* samples).

a

Figure 1b - Mouse embryogenesis

isoform	gen	source	cDNA	average	SD
Utrn 1A	wt	embyo day 7	3	187.44	29.53
Utrn 1A	wt	embyo day 11	3	336.08	23.38
Utrn 1A	wt	embyo day 15	3	31.68	10.67
Utrn 1A	wt	embyo day 17	3	12.59	2.82
Utrn 1A'	wt	embyo day 7	4	213.21	24.85
Utrn 1A'	wt	embyo day 11	4	89.10	13.10
Utrn 1A'	wt	embyo day 15	4	10.02	2.81
Utrn 1A'	wt	embyo day 17	4	10.93	1.85
Utrn 1B	wt	embyo day 7	4	89.09	10.67
Utrn 1B	wt	embyo day 11	4	90.97	8.35
Utrn 1B	wt	embyo day 15	4	21.20	3.90
Utrn 1B	wt	embyo day 17	4	16.62	1.96
Utrn 1B'	wt	embyo day 7	4	3.25	0.95
Utrn 1B'	wt	embyo day 11	4	11.46	1.58
Utrn 1B'	wt	embyo day 15	4	4.81	1.13
Utrn 1B'	wt	embyo day 17	4	3.96	1.10
Utrn 1C	wt	embyo day 7	4	16.62	0.28
Utrn 1C	wt	embyo day 11	4	69.49	5.51
Utrn 1C	wt	embyo day 15	4	7.75	2.21
Utrn 1C	wt	embyo day 17	4	4.35	1.69
Utrn 1D	wt	embyo day 7	2	4.36	na
Utrn 1D	wt	embyo day 11	2	10.33	na
Utrn 1D	wt	embyo day 15	2	1.98	na
Utrn 1D	wt	embyo day 17	2	3.49	na
Utrn 1F	wt	embyo day 7	3	12.69	8.46
Utrn 1F	wt	embyo day 11	3	108.59	15.64
Utrn 1F	wt	embyo day 15	3	18.37	6.86
Utrn 1F	wt	embyo day 17	3	14.86	3.13

b

Figure 1c - wild type (wt) expression in human foetal and adult tissue

isoform	gen	source	cDNA	average	SD
UTRN 1A	wt	emb. heart	4	13.12	0.59
UTRN 1A	wt	emb. lung	4	17.27	1.48
UTRN 1A	wt	emb. sk. musc.	4	7.11	0.31
UTRN 1A	wt	emb.thymus	4	21.75	1.19
UTRN 1A'	wt	emb. heart	3	7.41	0.34
UTRN 1A'	wt	emb. lung	3	9.05	1.01
UTRN 1A'	wt	emb. sk. musc.	3	4.08	0.59
UTRN 1A'	wt	emb.thymus	3	1.96	0.32
UTRN 1B	wt	emb. heart	3	7.08	0.38
UTRN 1B	wt	emb. lung	3	14.03	2.20
UTRN 1B	wt	emb. sk. musc.	3	2.87	0.31
UTRN 1B	wt	emb.thymus	3	63.06	2.23
UTRN 1B'	wt	emb. heart	3	3.17	1.04
UTRN 1B'	wt	emb. lung	3	8.41	0.43
UTRN 1B'	wt	emb. sk. musc.	3	6.52	0.72
UTRN 1B'	wt	emb.thymus	3	18.74	1.52
UTRN 1F	wt	emb. heart	3	4.19	0.57
UTRN 1F	wt	emb. lung	3	60.60	2.54
UTRN 1F	wt	emb. sk. musc.	3	4.34	0.28
UTRN 1F	wt	emb.thymus	3	16.81	0.92
UTRN 1A	wt	adult adipose	3	2.46	0.65
UTRN 1A	wt	adult bladder	3	0.74	0.43
UTRN 1A	wt	adult whole brain	3	1.87	0.19
UTRN 1A	wt	adult cervix	3	4.19	0.73
UTRN 1A	wt	adult colon	3	2.88	0.22
UTRN 1A	wt	adult esophagus	3	2.52	0.17
UTRN 1A	wt	adult heart	3	2.55	0.29
UTRN 1A	wt	adult kidney	3	3.49	0.34
UTRN 1A	wt	adult liver	3	0.29	0.24
UTRN 1A	wt	adult lung	3	3.97	0.23
UTRN 1A	wt	adult ovary	3	2.65	0.21
UTRN 1A	wt	adult placenta	3	1.51	0.01
UTRN 1A	wt	adult prostate	3	2.93	0.19
UTRN 1A	wt	adult sk. muscle	3	3.15	0.17
UTRN 1A	wt	adult smll intest.	3	1.58	0.58
UTRN 1A	wt	adult spleen	3	3.59	0.38
UTRN 1A	wt	adult testis	3	3.68	0.38
UTRN 1A	wt	adult thymus	3	4.00	0.53
UTRN 1A	wt	adult thyroid	3	3.19	0.41
UTRN 1A	wt	adult trachea	3	1.52	0.15

isoform	gen	source	cDNA	average	SD
UTRN 1A'	wt	adult adipose	3	1.07	0.26
UTRN 1A'	wt	adult bladder	3	0.45	0.32
UTRN 1A'	wt	adult whole brain	3	0.48	0.27
UTRN 1A'	wt	adult cervix	3	0.57	0.28
UTRN 1A'	wt	adult colon	3	2.12	0.19
UTRN 1A'	wt	adult esophagus	3	0.21	0.15
UTRN 1A'	wt	adult heart	3	1.53	0.16
UTRN 1A'	wt	adult kidney	3	2.19	0.32
UTRN 1A'	wt	adult liver	3	1.19	0.34
UTRN 1A'	wt	adult lung	3	2.96	0.40
UTRN 1A'	wt	adult ovary	3	1.26	0.15
UTRN 1A'	wt	adult placenta	3	1.10	0.40
UTRN 1A'	wt	adult prostate	3	4.10	0.12
UTRN 1A'	wt	adult sk. muscle	3	1.98	0.23
UTRN 1A'	wt	adult smll intest.	3	2.11	0.19
UTRN 1A'	wt	adult spleen	3	3.46	0.33
UTRN 1A'	wt	adult testis	3	2.63	0.37
UTRN 1A'	wt	adult thymus	3	0.22	0.48
UTRN 1A'	wt	adult thyroid	3	1.73	0.31
UTRN 1A'	wt	adult trachea	3	1.50	0.13
UTRN 1B	wt	adult adipose	3	2.37	0.27
UTRN 1B	wt	adult bladder	3	2.34	0.26
UTRN 1B	wt	adult whole brain	3	0.42	0.10
UTRN 1B	wt	adult cervix	3	1.99	0.32
UTRN 1B	wt	adult colon	3	0.64	0.16
UTRN 1B	wt	adult esophagus	3	0.77	0.37
UTRN 1B	wt	adult heart	3	2.70	0.34
UTRN 1B	wt	adult kidney	3	1.33	0.30
UTRN 1B	wt	adult liver	3	0.61	0.10
UTRN 1B	wt	adult lung	3	2.37	0.15
UTRN 1B	wt	adult ovary	3	2.95	0.43
UTRN 1B	wt	adult placenta	3	3.13	0.53
UTRN 1B	wt	adult prostate	3	1.11	0.26
UTRN 1B	wt	adult sk. muscle	3	0.82	0.04
UTRN 1B	wt	adult smll intest.	3	1.62	0.58
UTRN 1B	wt	adult spleen	3	7.09	0.15
UTRN 1B	wt	adult testis	3	0.99	0.38
UTRN 1B	wt	adult thymus	3	6.71	0.65
UTRN 1B	wt	adult thyroid	3	1.05	0.19
UTRN 1B	wt	adult trachea	3	0.59	0.25

isoform	gen	source	cDNA	average	SD
UTRN 1B'	wt	adult adipose	3	2.20	0.44
UTRN 1B'	wt	adult bladder	3	0.41	0.29
UTRN 1B'	wt	adult whole brain	3	0.34	0.21
UTRN 1B'	wt	adult cervix	3	2.23	0.14
UTRN 1B'	wt	adult colon	3	1.34	0.28
UTRN 1B'	wt	adult esophagus	3	1.55	0.39
UTRN 1B'	wt	adult heart	3	0.58	0.26
UTRN 1B'	wt	adult kidney	3	1.81	0.58
UTRN 1B'	wt	adult liver	3	0.72	0.31
UTRN 1B'	wt	adult lung	3	2.11	0.06
UTRN 1B'	wt	adult ovary	3	1.99	0.12
UTRN 1B'	wt	adult placenta	3	2.15	0.36
UTRN 1B'	wt	adult prostate	3	1.34	0.14
UTRN 1B'	wt	adult sk. muscle	3	2.39	0.17
UTRN 1B'	wt	adult smll intest.	3	2.55	0.11
UTRN 1B'	wt	adult spleen	3	8.63	0.40
UTRN 1B'	wt	adult testis	3	5.22	0.61
UTRN 1B'	wt	adult thymus	3	4.38	0.23
UTRN 1B'	wt	adult thyroid	3	1.90	0.70
UTRN 1B'	wt	adult trachea	3	1.10	0.34
UTRN 1F	wt	adult adipose	3	0.12	0.08
UTRN 1F	wt	adult bladder	3	0.37	0.08
UTRN 1F	wt	adult whole brain	3	0.35	0.10
UTRN 1F	wt	adult cervix	3	3.48	0.21
UTRN 1F	wt	adult colon	3	0.77	0.05
UTRN 1F	wt	adult esophagus	3	5.75	0.51
UTRN 1F	wt	adult heart	3	0.74	0.07
UTRN 1F	wt	adult kidney	3	3.69	0.43
UTRN 1F	wt	adult liver	3	0.27	0.04
UTRN 1F	wt	adult lung	3	5.97	0.12
UTRN 1F	wt	adult ovary	3	1.57	0.60
UTRN 1F	wt	adult placenta	3	6.81	0.83
UTRN 1F	wt	adult prostate	3	2.59	0.52
UTRN 1F	wt	adult sk. muscle	3	0.58	0.05
UTRN 1F	wt	adult smll intest.	3	0.14	0.11
UTRN 1F	wt	adult spleen	3	0.04	0.04
UTRN 1F	wt	adult testis	3	0.22	0.20
UTRN 1F	wt	adult thymus	3	1.97	0.15
UTRN 1F	wt	adult thyroid	3	7.68	0.45
UTRN 1F	wt	adult trachea	3	3.25	0.21

Supplementary Table 1: *Utrn/UTRN* qRT-PCR and sqRT-PCR values to accompany **Figure 1**.

Values obtained analysing utrophin isoform expression (**a**) during mouse embryogenesis and (**b**) human embryonic and adult tissue. For (**a**) and (**b**), columns are as follows: Isoform; mouse *Utrn* and human *UTRN* nomenclature to denote specific isoforms, gen; genotype (wt= wild type). Source; emb=embryonic, adult as denoted, abbreviations; sk. musc. / sk.muscle = skeletal muscle, smll intest. = small intestine. As data in **Figure 1** is represented in colour scale, the average of each sample is provided. Pooled total RNA was used, where “cDNA” column refers to number of individual reverse transcriptase reactions performed, with standard deviation calculated if n≥3. Each embryonic timepoint was subject to qRT-PCR in triplicate per cDNA preparation. For adult tissue, three cDNA preparations were used in individual sqRT-PCR reactions, quantified from separate agarose gels. Abbreviations, na = not applicable.

TABLE S2

a

Figure 2a- Mouse wild type and dystrophin-deficient tissue at postnatal 2 and 6 weeks

isoform	gen	source	br	average	SD	P-value	•		*t*-test vs.
Utrn 1A	wt	2wk diaph	4	147.21	26.24	-	-	-	-
Utrn 1A	*mdx*	2wk diaph	4	161.98	33.15	8.8E-02	-	-	2wk wt
Utrn 1A	wt	6wk diaph	4	99.73	22.36	-	-	-	-
Utrn 1A	*mdx*	6wk diaph	4	285.40	59.04	1.2E-07	•••	↑	6wk wt
Utrn 1A′	wt	2wk diaph	4	85.40	20.64	-	-	-	-
Utrn 1A′	*mdx*	2wk diaph	4	100.93	20.30	1.7E-03	•	↑	2wk wt
Utrn 1A′	wt	6wk diaph	4	62.81	14.18	-	-	-	-
Utrn 1A′	*mdx*	6wk diaph	4	201.30	47.79	4.0E-11	•••	↑	6wk wt
Utrn 1B	wt	2wk diaph	3	98.92	16.30	-	-	-	-
Utrn 1B	*mdx*	2wk diaph	3	135.32	19.83	5.5E-04	••	↑	2wk wt
Utrn 1B	wt	6wk diaph	3	85.73	9.40	-	-	-	-
Utrn 1B	*mdx*	6wk diaph	3	155.67	16.33	1.0E-05	•••	↑	6wk wt
Utrn 1B′	wt	2wk diaph	2	54.38	9.44	-	-	-	-
Utrn 1B′	*mdx*	2wk diaph	2	75.22	26.81	9.0E-02	-	-	2wk wt
Utrn 1B′	wt	6wk diaph	2	38.88	15.33	-	-	-	-
Utrn 1B′	*mdx*	6wk diaph	2	37.25	17.69	8.2E-01	-	-	6wk wt
Utrn 1C	wt	2wk diaph	2	3.27	1.17	-	-	-	-
Utrn 1C	*mdx*	2wk diaph	2	2.46	0.64	0.037	•	↓	2wk wt
Utrn 1C	wt	6wk diaph	2	2.96	0.97	-	-	-	-
Utrn 1C	*mdx*	6wk diaph	2	3.25	1.36	0.688	-	-	6wk wt
Utrn 1D	wt	2wk diaph	2	nd	-	-	-	-	-
Utrn 1D	*mdx*	2wk diaph	2	nd	-	-	-	-	-
Utrn 1D	wt	6wk diaph	2	nd	-	-	-	-	-
Utrn 1D	*mdx*	6wk diaph	2	nd	-	-	-	-	-
Utrn 1F	wt	2wk diaph	4	86.91	25.98	-	-	-	-
Utrn 1F	*mdx*	2wk diaph	4	145.95	41.23	4.2E-10	•••	↑	2wk wt
Utrn 1F	wt	6wk diaph	4	79.54	23.10	-	-	-	-
Utrn 1F	*mdx*	6wk diaph	4	80.50	23.94	8.2E-01	-	-	6wk wt
Utrn 1A	wt	2wk heart	4	266.55	43.81	-	-	-	-
Utrn 1A	*mdx*	2wk heart	4	339.72	30.53	5.0E-04	••	↑	2wk wt
Utrn 1A	wt	6wk heart	4	196.51	32.07	-	-	-	-
Utrn 1A	*mdx*	6wk heart	4	323.67	52.59	5.2E-07	•••	↑	6wk wt
Utrn 1A′	wt	2wk heart	3	175.74	19.80	-	-	-	-
Utrn 1A′	*mdx*	2wk heart	3	204.21	37.90	9.4E-02	-	-	2wk wt
Utrn 1A′	wt	6wk heart	3	169.24	27.13	-	-	-	-
Utrn 1A′	*mdx*	6wk heart	3	208.01	53.37	3.3E-02	-	-	6wk wt
Utrn 1B	wt	2wk heart	4	267.22	30.14	-	-	-	-
Utrn 1B	*mdx*	2wk heart	4	297.31	41.00	0.0853	-	-	2wk wt
Utrn 1B	wt	6wk heart	4	225.21	30.65	-	-	-	-
Utrn 1B	*mdx*	6wk heart	4	334.52	37.56	1.4E-04	••	↑	6wk wt
Utrn 1B′	wt	2wk heart	2	168.51	na	na	-	-	-
Utrn 1B′	*mdx*	2wk heart	2	179.76	na	na	-	-	-
Utrn 1B′	wt	6wk heart	2	171.97	na	na	-	-	-
Utrn 1B′	*mdx*	6wk heart	2	173.47	na	na	-	-	-
Utrn 1C	wt	2wk heart	2	14.23	na	na	-	-	-
Utrn 1C	*mdx*	2wk heart	2	11.23	na	na	-	-	-
Utrn 1C	wt	6wk heart	2	8.91	na	na	-	-	-
Utrn 1C	*mdx*	6wk heart	2	10.44	na	na	-	-	-
Utrn 1D	wt	2wk heart	2	1.39	na	na	-	-	-
Utrn 1D	*mdx*	2wk heart	2	2.14	na	na	-	-	-
Utrn 1D	wt	6wk heart	2	0.72	na	na	-	-	-
Utrn 1D	*mdx*	6wk heart	2	1.53	na	na	-	-	-
Utrn 1F	wt	2wk heart	3	28.14	7.04	-	-	-	-
Utrn 1F	*mdx*	2wk heart	3	29.34	3.08	0.591	-	-	2wk wt
Utrn 1F	wt	6wk heart	3	29.30	3.45	-	-	-	-
Utrn 1F	*mdx*	6wk heart	3	38.00	10.09	0.050	-	-	6wk wt
Utrn 1A	wt	2wk lung	4	406.46	53.88	-	-	-	-
Utrn 1A	*mdx*	2wk lung	4	491.46	72.44	4.9E-03	•	↑	2wt wt
Utrn 1A	wt	6wk lung	4	410.69	34.98	-	-	-	-
Utrn 1A	*mdx*	6wk lung	4	865.87	95.70	1.3E-08	•••	↑	6wk wt
Utrn 1A′	wt	2wk lung	4	97.66	16.74	-	-	-	-
Utrn 1A′	*mdx*	2wk lung	4	115.98	21.12	8.4E-03	•	↑	2wk wt
Utrn 1A′	wt	6wk lung	4	94.76	22.65	-	-	-	-
Utrn 1A′	*mdx*	6wk lung	4	191.18	40.97	4.1E-06	•••	↑	6wk wt
Utrn 1B	wt	2wk lung	4	219.33	60.38	-	-	-	-
Utrn 1B	*mdx*	2wk lung	4	208.39	49.54	6.7E-01	-	-	2wk wt
Utrn 1B	wt	6wk lung	4	210.97	52.80	-	-	-	-
Utrn 1B	*mdx*	6wk lung	4	306.76	75.11	5.3E-03	•	↑	6wk wt
Utrn 1B′	wt	2wk lung	3	131.46	28.80	-	-	-	-
Utrn 1B′	*mdx*	2wk lung	3	159.94	28.50	1.1E-01	-	-	2wk wt
Utrn 1B′	wt	6wk lung	3	78.37	10.74	-	-	-	-
Utrn 1B′	*mdx*	6wk lung	3	178.43	32.11	4.9E-03	•	↑	6wk wt
Utrn 1C	wt	2wk lung	2	28.98	na	na	-	-	-
Utrn 1C	*mdx*	2wk lung	2	31.03	na	na	-	-	-
Utrn 1C	wt	6wk lung	2	33.58	na	na	-	-	-
Utrn 1C	*mdx*	6wk lung	2	34.22	na	na	-	-	-
Utrn 1D	wt	2wk lung	1	1.20	na	na	-	-	-
Utrn 1D	*mdx*	2wk lung	1	0.98	na	na	-	-	-
Utrn 1D	wt	6wk lung	1	0.82	na	na	-	-	-
Utrn 1D	*mdx*	6wk lung	1	0.90	na	na	-	-	-
Utrn 1F	wt	2wk lung	3	66.87	16.49	-	-	-	-
Utrn 1F	*mdx*	2wk lung	3	65.62	12.44	7.9E-01	-	-	2wk wt
Utrn 1F	wt	6wk lung	3	28.63	8.75	-	-	-	-
Utrn 1F	*mdx*	6wk lung	3	34.47	8.84	2.5E-01	-	-	6wk wt
Utrn 1A	wt	2wk hind	4	30.33	6.82	-	-	-	-
Utrn 1A	*mdx*	2wk hind	4	59.77	10.70	4.3E-07	•••	↑	2wk wt
Utrn 1A	wt	6wk hind	3	31.13	6.54	-	-	-	-
Utrn 1A	*mdx*	6wk hind	3	45.79	6.17	4.4E-07	•••	↑	6wk wt
Utrn 1A′	wt	2wk hind	3	14.40	3.38	-	-	-	-
Utrn 1A′	*mdx*	2wk hind	3	23.19	2.47	5.7E-04	••	↑	2wk wt
Utrn 1A′	wt	6wk hind	3	13.64	3.05	-	-	-	-
Utrn 1A′	*mdx*	6wk hind	3	21.59	2.21	1.2E-05	••	↑	6wk wt
Utrn 1B	wt	2wk hind	2	32.56	na	na	-	-	-
Utrn 1B	*mdx*	2wk hind	2	31.26	na	na	-	-	-
Utrn 1B	wt	6wk hind	2	31.62	na	na	-	-	-
Utrn 1B	*mdx*	6wk hind	2	36.77	na	na	-	-	-
Utrn 1B′	wt	2wk hind	2	12.25	na	na	-	-	-
Utrn 1B′	*mdx*	2wk hind	2	19.87	na	na	-	-	-
Utrn 1B′	wt	6wk hind	2	13.25	na	na	-	-	-
Utrn 1B′	*mdx*	6wk hind	2	23.22	na	na	-	-	-
Utrn 1C	wt	2wk hind	2	1.38	na	na	-	-	-
Utrn 1C	*mdx*	2wk hind	2	1.10	na	na	-	-	-
Utrn 1C	wt	6wk hind	2	1.57	na	na	-	-	-
Utrn 1C	*mdx*	6wk hind	2	2.00	na	na	-	-	-
Utrn 1D	wt	2wk hind	2	nd	-	-	-	-	-
Utrn 1D	*mdx*	2wk hind	2	nd	-	-	-	-	-
Utrn 1D	wt	6wk hind	2	nd	-	-	-	-	-
Utrn 1D	*mdx*	6wk hind	2	nd	-	-	-	-	-
Utrn 1F	wt	2wk hind	4	15.06	4.15	-	-	-	-
Utrn 1F	*mdx*	2wk hind	4	34.91	7.10	7.3E-06	•••	↑	2wk wt
Utrn 1F	wt	6wk hind	4	16.79	3.23	-	-	-	-
Utrn 1F	*mdx*	6wk hind	4	70.21	10.08	2.2E-09	•••	↑	6wk wt

b

Figure 2b - Mouse C2C12 myogenesis

isoform	gen	source	wells	average	SD
Utrn 1A	wt	C2C12 myoblast	4	318.98	25.44
Utrn 1A	wt	C2C12 diff day 3	4	878.65	79.52
Utrn 1A	wt	C2C12 diff day 6	4	725.63	94.49
Utrn 1A	wt	C2C12 diff day 9	4	724.22	97.57
Utrn 1A′	wt	C2C12 myoblast	4	251.40	25.45
Utrn 1A′	wt	C2C12 diff day 3	4	559.87	64.81
Utrn 1A′	wt	C2C12 diff day 6	4	555.75	58.69
Utrn 1A′	wt	C2C12 diff day 9	4	537.65	59.39
Utrn 1B	wt	C2C12 myoblast	2	5.18	na
Utrn 1B	wt	C2C12 diff day 3	2	4.76	na
Utrn 1B	wt	C2C12 diff day 6	2	4.53	na
Utrn 1B	wt	C2C12 diff day 9	2	4.95	na
Utrn 1B′	wt	C2C12 myoblast	2	3.43	na
Utrn 1B′	wt	C2C12 diff day 3	2	2.00	na
Utrn 1B′	wt	C2C12 diff day 6	2	2.40	na
Utrn 1B′	wt	C2C12 diff day 9	2	2.61	na
Utrn 1C	wt	C2C12 myoblast	2	34.82	na
Utrn 1C	wt	C2C12 diff day 3	2	46.66	na
Utrn 1C	wt	C2C12 diff day 6	2	49.38	na
Utrn 1C	wt	C2C12 diff day 9	2	49.96	na
Utrn 1D	wt	C2C12 myoblast	2	2.49	na
Utrn 1D	wt	C2C12 diff day 3	2	2.72	na
Utrn 1D	wt	C2C12 diff day 6	2	2.64	na
Utrn 1D	wt	C2C12 diff day 9	2	2.24	na
Utrn 1F	wt	C2C12 myoblast	4	101.03	17.84
Utrn 1F	wt	C2C12 diff day 3	4	142.26	20.34
Utrn 1F	wt	C2C12 diff day 6	4	160.00	25.37
Utrn 1F	wt	C2C12 diff day 9	4	168.89	22.40

Supplementary Table 2: *Utrn qRT-PCR* values and statistics to accompany **Figure 2**.

Values and statistics for utrophin isoform expression in (**a**) 2-/6-week C57/BL10 control and *mdx*/BL10 mouse tissue and (**b**) during myogenic differentiation of C2C12 cells. As data in **Figure 2** is represented in colour scale, the average, standard deviation (SD) and relevant statistical analysis of each sample is provided here. For (**a**) columns are as follows: Isoform; mouse *Utrn* nomenclature to denote specific isoforms, gen; genotype (wt=wild type; *mdx*=dystrophin-deficient), Source; week and tissue as indicated; diaphrm; diaphragm, nd = not detected above –RT, wk = week, na = not applicable (standard deviation and P-values were not calculated for samples with n<3 biological repeats). Two tailed t-tests were perfomed against a second “control” sample as indicated, P-values (99% cl) were assigned significance by the following symbols: P<0.0001••• P<0.001••, P<0.05•; with commensurate increase / decrease denoted by up green and down red arrows respectively. The number of mice used per isoform / timepoint is stated in column “br” (biological replicate). For (**b**) abbreviations as follows: diff = differentation, na = not applicable, wells = number of separate, non-pooled wells used to synthesise cDNA. Standard deviation was calculated if n>3 wells were used. All qRT-PCR reactions in (**a**) and (**b**) were performed in triplicate. Average and standard deviation (SD) are provided to the second decimal place.
